# Gene-diet interactions and cardiovascular diseases: a systematic review of observational and clinical trials

**DOI:** 10.1186/s12872-022-02808-1

**Published:** 2022-08-20

**Authors:** Zayne M. Roa-Díaz, Julian Teuscher, Magda Gamba, Marvin Bundo, Giorgia Grisotto, Faina Wehrli, Edna Gamboa, Lyda Z. Rojas, Sergio A. Gómez-Ochoa, Sanne Verhoog, Manuel Frias Vargas, Beatrice Minder, Oscar H. Franco, Abbas Dehghan, Raha Pazoki, Pedro Marques-Vidal, Taulant Muka

**Affiliations:** 1 Institute of Social and Preventive Medicine (ISPM), University of Bern, Mittelstrasse 43, 3012 Bern, Switzerland; 2 Graduate School for Health Sciences, University of Bern, Bern, Switzerland; 3 Oeschger Center for Climate Change Research, University of Bern, Bern, Switzerland; 4 Department of Nutrition, Harvard T.H. Chan School of Public Health, Boston, MA USA; 5 School of Nutrition and Dietetics, Health Faculty, Universidad Industrial de Santander, Bucaramanga, Colombia; 6 Nursing Research and Knowledge Development Group GIDCEN, Fundación Cardiovascular de Colombia, Floridablanca, Colombia; 7 Department of Public Health, Erasmus MC, University Medical Center Rotterdam, Rotterdam, The Netherlands; 8 Centro de Salud Universitario Comillas, Madrid, Spain; 9 Public Health & Primary Care Library, University Library of Bern, University of Bern, Bern, Switzerland; 10 Department of Epidemiology, Erasmus MC University Medical Center, Rotterdam, The Netherlands; 11 Department of Life Sciences, College of Health and Life Sciences, Brunel University London, Uxbridge, UK; 12 Department of Epidemiology and Biostatistics, MRC Centre for Environment and Health, School of Public Health, Imperial College London, London, UK; 13 CIRTM Centre for Inflammation Research and Translational Medicine, College of Health and Life Sciences, Brunel University London, Uxbridge, UK; 14 Department of Medicine, Internal Medicine, Lausanne University Hospital (CHUV) and University of Lausanne, Lausanne, Switzerland

**Keywords:** Diet, Gene-diet interaction, Myocardial infarction, Stroke, Coronary heart disease, Cardiovascular diseases

## Abstract

**Background:**

Both genetic background and diet are important determinants of cardiovascular diseases (CVD). Understanding gene-diet interactions could help improve CVD prevention and prognosis. We aimed to summarise the evidence on gene-diet interactions and CVD outcomes systematically.

**Methods:**

We searched MEDLINE^®^ via Ovid, Embase, PubMed^®^, and The Cochrane Library for relevant studies published until June 6th 2022. We considered for inclusion cross-sectional, case–control, prospective cohort, nested case–control, and case-cohort studies as well as randomised controlled trials that evaluated the interaction between genetic variants and/or genetic risk scores and food or diet intake on the risk of related outcomes, including myocardial infarction, coronary heart disease (CHD), stroke and CVD as a composite outcome. The PROSPERO protocol registration code is CRD42019147031.

**Results and discussion:**

We included 59 articles based on data from 29 studies; six articles involved multiple studies, and seven did not report details of their source population. The median sample size of the articles was 2562 participants. Of the 59 articles, 21 (35.6%) were qualified as high quality, while the rest were intermediate or poor. Eleven (18.6%) articles adjusted for multiple comparisons, four (7.0%) attempted to replicate the findings, 18 (30.5%) were based on Han-Chinese ethnicity, and 29 (49.2%) did not present Minor Allele Frequency. Fifty different dietary exposures and 52 different genetic factors were investigated, with alcohol intake and ADH1C variants being the most examined. Of 266 investigated diet-gene interaction tests, 50 (18.8%) were statistically significant, including CETP-TaqIB and ADH1C variants, which interacted with alcohol intake on CHD risk. However, interactions effects were significant only in some articles and did not agree on the direction of effects. Moreover, most of the studies that reported significant interactions lacked replication. Overall, the evidence on gene-diet interactions on CVD is limited, and lack correction for multiple testing, replication and sample size consideration.

**Supplementary Information:**

The online version contains supplementary material available at 10.1186/s12872-022-02808-1.

## Introduction

Cardiovascular diseases (CVDs), including ischemic heart disease and stroke, are the leading cause of mortality and morbidity and are responsible for more than 18 million deaths globally in 2019 [1]. Several risk factors have been associated with CVD incidence, diet being one of the most studied [2].

Contradictory findings have been reported on the role of micro-and macro-nutrients [3], specific foods [4], and dietary patterns [5] on CVD. These contradictions could be explained by the exclusion of genetic factors [6], which has a causal association with CVD onset [7–9]. Therefore, studying the combined impact of food intake/dietary patterns and genetic risk on CVD may improve CVD prevention and care precision [10]. Several studies have shown dietary components such as carbohydrates, micronutrients, vegetables, fatty acids, and alcohol to be linked with different genetic factors on CVD [11–17]. However, no systematic review summarising the evidence on diet-gene interaction on CVD has been published to date.

Previous systematic reviews published on the topic have primarily focused on evaluating gene-diet interactions on specific genes or have been restricted to particular dietary groups [18, 19]. In addition, understanding the association between pathological pathway factors requires distinguishing between statistical and biological interactions. In the context of gene-environment interaction (GxE), statistical interaction is understood as a deviation from the additivity of the effects of two exposures (genetic and environmental) on the outcome. In contrast, biological interactions are defined as the co-participation of two exposures in the same causal mechanism for the development of the outcome, regardless of their statistical ascertainment [20]. This paper focuses on statistical interactions, more frequently tested in the epidemiological literature [21]. Identifying exposure-disease interactions may help recognise groups at increased risk due to genetic susceptibility and help tailor prognostic tools and intervention strategies [22]. Therefore, we aimed to systematically summarise the evidence on gene-diet interactions and cardiovascular disease risk: CHD, myocardial infarction (MI), stroke, and CVD as a composite outcome.

## Methods

The protocol of this systematic review was registered in PROSPERO (https://www.crd.york.ac.uk/prospero/dayisplay_record.php?ID=CRD42019147031). For the conduct and reporting of this systematic review, we followed the steps proposed by Muka et al. [23] and Synthesis without meta-analysis (SWiM) in systematic reviews: reporting guideline [24].

### Literature search

Studies were primarily identified through structured searches in MEDLINE^®^ via Ovid, Embase, PubMed^®^, and The Cochrane Library, where we were searched for articles published until June 6th 2022 without language restriction. The search strategy was designed and implemented in collaboration with an experienced medical librarian (BM). This search strategy was designed based on subject headings (e.g. MeSH terms) and free text words related to three search domains: (1) diet, food, nutrition, (2) gene-diet interaction, and (3) cardiovascular diseases. Additional file 1: Appendix S1 contains the complete search strategies.

### Study selection criteria

Studies conducted in the adult population were eligible for inclusion if (i) they were cross-sectional, case–control, prospective cohort, nested case–control or case-cohort studies, or randomised controlled trials; (ii) evaluated dietary intakes (micro- and macro-nutrients, specific food items, food groups, dietary scores, indexes, or patterns) Additional file 2: Table S1 [25]; (iii) evaluated incident or prevalent CVD as a composite outcome or any of the following outcomes: CHD, MI or stroke; (iv) evaluated the interaction between any genetic variant or genetic risk score (GRS) and food or diet intake; and (v) reported a statistical test for gene-diet interaction. We excluded epigenetic studies and publications that did not report a statistical test and p-values for the interaction between diet and genetics. Abstracts, cost-effectiveness studies, letters to the editor, conference proceedings, systematic reviews and meta-analyses were excluded.

### Screening and study selection

All studies initially identified were screened independently by two authors applying the selection criteria. After that, the full texts of the studies that met the selection criteria were further evaluated independently by two authors. When there were discrepancies, the two authors reached a consensus or asked for the help of a third senior author.

### Data extraction

Information from the included articles was registered in a pre-designed form; the data were first extracted by the first author and additionally reviewed and confirmed by a second author. We collected the author's name, year of publication, country of origin of the population, ethnicity, setting, study design, name of the cohort, sample size, number of cases (CVD as a composite outcome, CHD, MI, or stroke), definition of the reported cases, percentage of women included, follow-up time, dietary intake evaluated, dietary intake measurements, genes, genetic variants assessed, minor allele frequency (MAF), and main findings. The estimates and p-values for gene-diet interactions were taken from the most adjusted model.

### Assessing the quality of studies

We applied a quality score designed for gene-diet interaction studies [26]. The score evaluates eight items: interaction as primary study goal, test for interaction, correction for multiple testing, correction for ethnicity, Hardy‐Weinberg equilibrium, test for group similarity at baseline, sample size, and sufficient details of the study procedure. Based on a range scale from − 8 to 8, studies were rated as high quality (6 to 8 points), intermediate quality (2 to 5 points), and poor quality (− 8 to 1 point). All the included studies were treated equally regardless of their quality.

### Synthesis methods

A meta-analysis could not be carried out given the diversity of dietary exposures, gene-diet interactions, and the methodological heterogeneity of the included studies (different dietary exposures, gene variants and assessed interactions). We summarised the gene-diet interactions finding qualitatively and decided to group the included studies in two stages. First, we grouped the studies according to the assessed outcome into the following categories: CHD, stroke, and CVD as a composite outcome. Second, we presented the gene-diet interaction information according to five dietary intake groups (macronutrients, micronutrients, food and food items categories, other dietary components and dietary scores, indexes, or patterns) Additional file 2: Table S1 [25].

The principal characteristics and findings of the included studies are presented in tabular format. Additionally, we represented the interaction between dietary intake groups and genetic variants with CHD, stroke, and CVD through a heat map where *p*-values of diet-gene interactions are represented by colour intensity where the lowest *p* values have the most intense colour, and values near 1 have the lightest colour. All heat maps were created in R software environment for statistical computing [27] with RStudio environment [28] using the ggplot2 package [29]. To standardise the amount/frequency of alcohol intake reported in the interaction with an alcohol dehydrogenase 1C (ADH1C) variant, we transformed grams/day into drinks/week taking as reference the "standard" drink (14 g of pure alcohol) reported by the National Institute on Alcohol Abuse and Alcoholism (NIAAA) [30].

## Results

### Study identification and selection

**Fig. 1:**
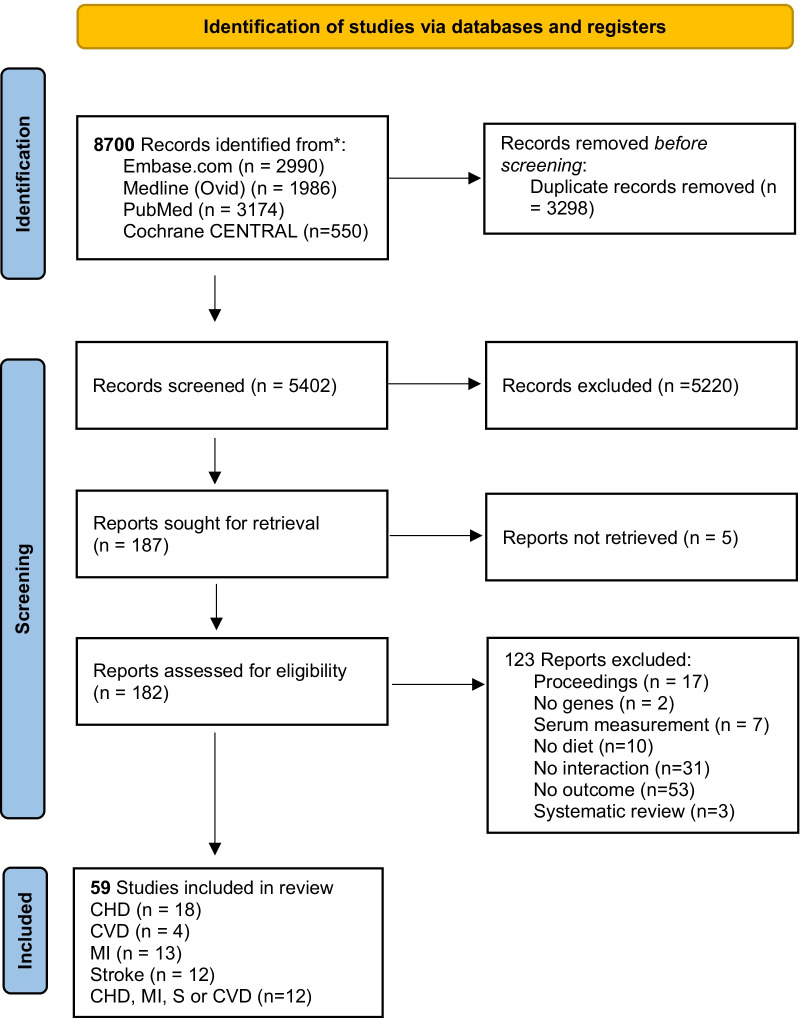
Flow chart of study selection

We identified 8700 articles, of which 5402 were unique citations. After screening titles and abstracts, we screened the full texts of 182 articles, of which 59 met the inclusion criteria and were included in the final analysis (Fig. 1). Of the included articles, 13 evaluated MI [16, 31–42], 18 evaluated CHD [11–13, 43–57], 12 evaluated stroke [14, 58–68], four examined composite CVD [15, 69–71], and 12 evaluated at least two of the following outcomes: CHD, MI, CVD or stroke [6, 17, 72–81]. The definition of all outcomes can be found in Additional file 2: Table S2.

### Characteristics of all included studies and articles reporting significant gene-diet interactions

The general characteristics are described in terms of number of articles. Forty-five articles came from 29 unique studies; six articles involved multiple studies, and seven did not report details of their source population. Of the 59 articles, 24 (40.7%) were conducted in Europe, 21 (35.6%) in China, six (10.2%) in the USA, five (8.5%) in Costa Rica, one (1.7%) in Taiwan, one (1.7%) in Thailand and one (1.7%) was multicentre. The ethnicity most frequently reported was Chinese-Han in 18 (30.5%) articles, followed by Caucasian in eight (13.6%) articles, Hispanic/Latin American in five (8.5%) articles, and Mediterranean in four (6.8%) articles. The epidemiological designs of the included articles comprised 27 (45.8%) case–control studies, 19 (32.2%) prospective cohort studies, seven (11.9%) nested case–control studies, one (1.7%) case-cohort study, two (3.3%) randomised control trial studies, two (3.3%) family-based studies, and one (1.7%) cross-sectional study. The median sample size in the articles was 2562, ranging from 200 to 347,077 participants. Men and women were analysed in 53 (89.8%) articles; five (8.5%) articles analysed only men, and one (1.7%) article only women. The main interaction results among female study participants were presented in ten (17.0%) articles. The median age of participants among studies was 61 years, ranging from 57 to 72.4 (Table 1).

**Table 1:** Study characteristics

Reference	Country (Ethnicity)	Study Type (Recruitment setting)	Cohort name (FU years)	No. of participants (cases/total)	Sex	Interactor diet (Type of measurement)	(Gene/chromosome region) and (SNP/GRS)	Significant interactions	Replication
*Coronary heart disease—myocardial infarction*									
Allayee H et al. [31]	Costa Rica (Hispanic)	Case–Control (Population)	–	1885/3770	Both	Arachidonic acid intake (Questionnaire)	5-LO (33–37; 44–46; 48; 55–59; 66; 67)	Yes	No
Chen Q et al. [32]	China (Han-Chinese)	Case–Control (Population)	–	300/600	Both	Alcohol (Standardized questionnaires)	PCSK9 (rs11206510)	Yes	No
Cornelis M et al., 2007 [33]	Costa Rica (Hispanic)	Case–Control (Population)	–	2042/4084	Both	Cruciferous vegetables (FFQ)	GSTT1, GSTP1, GSTM1(-)	Yes	No
Cornelis M et al. [34]	Costa Rica (Hispanic)	Case–Control (Population)	–	2014/4028	Both	Coffee (Questionnaire)	CYP1A2 (rs762551)	Yes	No
Ding Y et al., 2016 [16]	Norway (European)	Cohort (Clinical)	WENBIT (5)	206/2381	Both	Vitamin B12 and vitamin B6 (According to the cohort data)	MTHFD1 (rs1076991)	Yes	No
Fumeron F et al. [35]	France (White Europeans)	Case–Control (Population)	ECTIM Etude Cas-Témoin de l'Infarctus du Myocarde(NR)	608/1332	Men	Alcohol (Questionnaire)	CETP (rs708272 (CETP/TaqIB))	Yes	No
Hartiala J et al. [36]	Costa Rica (Latin American)	Case–Control (Clinical)	–	1936/3971	Both	PUFAs (Polyunsaturated fatty acids) (Questionnaire)	PLA2G4A (rs12746200)	Yes	Yes
Hines L et al. [37]	United States (not described)	nested case–Control (Population)	Physicians’ Health Study (NR)	396/1166	Men	Alcohol (Questionnaire)	ADH1C (rs698)	Yes	No
Li J et al. [38]	China (Han-Chinese)	Case–Control (Population)	–	344/688	Both	Alcohol (Standardised questionnaire)	CONNEXIN 37 (rs1764391)	Yes	No
Tolstrup J et al. [39]	Denmark (Danish general population)	Cohort (Population)	CCHS (16)	663/9584	Both	Alcohol (Questionnaire)	ADH1C/ ADH1B (rs698, rs1229984)	No	No
Trichopolou A et al. [40]	Greece (Mediterranean)	Nested Case–Control (Population)	Greek—EPIC(NR)	202/399	Both	Mediterranean diet (Questionnaire)	APOA5, APOC3, APOE, IL1β, IL6, LPL, MTHFR, NOS3, and TNF (GRS-MI (rs429358, rs7412, rs662799, rs5128, rs1801177, rs268, rs328, rs1801133, rs1799983, rs16944, rs1800795, rs1800629))	No	No
Wang F et al. [41]	China (Han-Chinese)	Case–Control (Population)	–	300/600	Both	Alcohol (Interview on alcohol intake in the last 12 months)	CXCL12 (rs1746048)	Yes	No
Zheng Y et al. [42]	Costa Rica (Hispanic)	Case–Control (Population)	–	1560/3311	Both	Sugar-sweetened beverages (Questionnaire)	CDKN2B-AS1 (GRS (rs4977574, rs2383206, rs1333049))	Yes	No
*Coronary heart disease*									
Bos M M et al. [57]	United Kingdom (not described)	Cohort (Population)	UK Biobank	12,806/345659	Both	Oily fish intake (Questionnaire)	APOE(-)	No	No
Chen H et al. [43]	China (Han-Chinese)	Case–Control (Clinical)	–	429/751	Both	Alcohol (Self-reported)	Il6 (rs1800795, rs1800796, rs1800797)	Yes	No
Chi Y et al. [44]	China (Han-Chinese)	Case–Control (Clinical/ Population)	–	631/1269	Both	Alcohol (Questionnaire)	PLA2G7 (rs1805018, rs16874954, rs1805017 and rs1051931)	No	No
Corella D et al. [45]	Spain (Mediterranean)	Nested Case–Control (Population)	Spanish EPIC (10)	557/1737	Both	Alcohol (Questionnaire)	CETP (rs708272 (CETP/TaqIB))	Yes	No
Ebrahim S et al. [11]	United Kingdom (not described)	Cohort (Population)	BWHHS and Caerphilly cohorts (NR)	283/4547	Both	Alcohol (Questionnaire)	ADH1C (rs1693482)	No	Yes
Gustavsson J et al. [46]	Sweden (not described)	Case–Control (Population)	SHEEP and INTERGENE	1381/5671	Both	PUFA, SFA, Carbohydrates, Sucrose, Protein, Fat (semi-quantitative FFQ)	FTO (rs9939609)	No	No
Heidrich J et al., 2007 [47]	Germany (Caucasian)	Cohort (Population)	MONICA-KORA project (7.8)	72/3664	Both	Alcohol (Interview)	ADH1C (rs698)	Yes	No
Huang L et al. [48]	China (Han)	Nested Case–Control (Population)	Yinzhou District of Ningbo, Zhejiang Province, China (3)	161/656	Both	Dessert and fried food (Questionnaire)	ALDH2 (rs671)	No	No
Jensen M et al., 2008 [12]	United States (not described)	Nested Case–Control (Population)	NHS and HPFS (NR)	505/1504	Both	Alcohol (Questionnaire)	CETP (rs708272 (CETP/TaqIB))	Yes	Yes
Liu F et al. [49]	China (not described)	Case–Control (Clinical)	–	838/1278	Both	n-3 Polyunsaturated Fatty Acid (n-3 LCPUFA) (Questionnaire)	FADS1 (rs174547)	Yes	No
Liu Y et al. [50]	Taiwan (not described)	Case–Control (Population)	TWB (NR)	1116/8969	Both	Coffee (Interview on coffee intake in the last 6 months, regular intake defined as 3 or more cups of coffee/week)	TRIB1 (rs17321515)	Yes	No
Mehlig K et al. [51]	Sweden (not described)	Case–Control (Population)	INTERGENE	618/3539	Both	Alcohol (Interview)	CETP (rs708272 (CETP/TaqIB))	Yes	No
Tolstrup J et al. [13]	Denmark (Caucasian)	Nested Case-Cohort (Population)	Danish Diet, Cancer and Health Cohort (NR)	770/1645	Men	Alcohol (FFQ)	ADH1B/ADH1C (rs1229984/rs1693482)	No	No
Virtanen J et al. [53]	Finland (not described)	Cohort (Population)	KIHD (20.8)	230/1032	Men	Egg/ Cholesterol (Guided 4-d food records)	APOE4 (E2/2, E2/3, E2/4, E3/3, E3/4 and E4/4)	No	No
Yiannakouris N et al. [54]	Greece (European)	Nested Case–Control (Population)	Greek-EPIC (10)	477/1748	Both	Mediterranean diet (Questionnaire)	PCSK9, CELSR2-PSRC1-SORT1, MIA3, WDR12, PHACTR1, CXCL12, LDLR, SLC5A3-MRPS6-KCNE2, CDKN2A/2B (GRS-CHD (rs11206510, rs646776, rs17465637, rs6725887, rs9349379, rs1746048, rs1122608, rs9982601 and rs1333049))	No	No
Younis J et al. [55]	United Kingdom (not described)	Cohort (Clinical)	NPHS II(NR)	220/2773	Men	Alcohol (Questionnaire)	ADH1C (^γ1^ ^γ1^, ^γ1^ ^γ2^, ^γ2^ ^γ2^)	No	No
Zhou H et al. [56]	China (Han-Chinese)	Case–Control (Clinical)	–	610/1833	Both	Alcohol (Questionnaire filled out in face-to-face interviews)	TFPI-2 (rs59805398, rs34489123, rs4264, rs4271)	No	No
Mukamal K et al. [52]	United States (American)	Nested Case–Control (Population)	NHS and HPFS (7)	506/1524	Both	Alcohol (Questionnaire)	PON1	No	No
*Stroke*									
Mukamal K et al. [62]	United States (not described)	Cohort (Population)	CHS Cardiovascular Health Study (9.2)	434/4410	Both	Alcohol (Questionnaire)	APOE (-)	No	No
Chen Z et al. [58]	China (Han-Chinese)	Case–Control (Population)	–	159/334	Both	Alcohol (Questionnaire)	CRP (rs1800947, rs3093059)	Yes	No
Gao X et al. [59]	China (Han-Chinese)	Case–Control (Clinical)	–	100/200	Both	Alcohol (Questionnaire)	FgB (FgBCT/TT)	Yes	No
Juan J et al. [60]	China (not described)	Family-based case–control-study (Clinical/ Population)	FISSIC (NR)	1007/2158	Both	Vegetable and fruit intake (Semi-quantitative FFQin face-to-face survey)	PON1 (rs662)	No	No
Zhou YG et al. [68]	China (Han-Chinese)	Case–control (Clinical)	–	544/1005	Both	Alcohol (Questionnaire)	(DGAT2 rs11236530, DGAT2 rs3060, MOGAT2 rs600626, MOGAT2 rs609379, and MOGAT2 rs10899104)	No	No
Kamdee K et al. [67]	Thailand (Thai-Buddhist)	Case–Control (Clinical)	–	200/400	Both	Alcohol (medical records)	IL-6 (rs1800795) and TNF-α (rs1800629)	Yes	No
Luo S et al. [61]	China (Han-Chinese)	Case–Control (Clinical)	–	308/602	Both	Alcohol (Medical history)	Il8 (Il8 + 781 C/T)	No	No
Song J et al. [63]	China (not described)	Family-based-cohort-study (Population)	FISSIC	1213/5869	Both	Eggs (Semi-quantitative FFQ)	ABCA1 (rs2066715)	No	No
Yang S et al. [14]	China (Han-Chinese)	Case–Control and cohort	-(5)	2012/4222	Both	Alcohol (Interview)	ACTB (rs852426, rs852423 and rs2966449)	Yes	Yes
Zhang L et al. [64]	China (Han-Chinese)	Case–Control (Clinical)	–	881/1773	Both	Alcohol (Interview)	CONNEXIN 37 and PDE4D (rs1764391, rs1764390, rs918592 and rs966220)	No	No
Zhao T et al. [65]	China (Han-Chinese)	Case–Control (Population/ Clinical)	–	161/644	Both	Fruits/ Vegetables (Semi-quantitative FFQ)	BCO2(rs10431036) BCO2(rs11214109) TRIB1(rs17321515) TRIB1(rs2954029)	No	No
Zheng X et al. [66]	China (Han-Chinese)	Case–Control (Population)	–	860/1722	Both	Alcohol (NR)	MTHFR (rs4846049, rs1537514, rs3737967, and rs4846048)	Yes	No
*Cardiovascular disease*									
Djousse L et al. [69]	United States (not described)	Cohort (Population)	Framingham Heart Study (NR)	132/1805	Both	Alcohol (Interview)	ADH1C (rs698, rs1693482)	No	No
Hindy G et al. [70]	Sweden (Caucasian)	Cohort (Population)	MDCS (15)	3164/23949	Both	Vegetable, fruit, wine, alcohol (Diet history)	9p21 locus(rs4977574)	Yes	No
Sonestedt S et al. [15]	Sweden (not described)	Cohort (Population)	MDCS (14)	2921/26455	Both	Sucrose, fibre, vegetables, fruits and berries, juice, potatoes, whole grains, refined grains, cookies and cakes, sugar and sweets, sugar-sweetened beverages (Questionnaire)	-(GRS-dyslipidaemia (26 SNPs for triglycerides, 41 SNPs for HDL-C and 32 SNPs for LDL-C))	No	No
Zhou A et al. [71]	United Kingdom (not described)	Cohort (Population)	UK Biobank (NR)	8368/347077	Both	Coffee (Interview)	CYP1A2 (rs762551/ GRS for metabolism of caffeine (rs4410790, rs6968554, rs10275488, rs2892838, rs12909047, rs35107470, rs2470893, and rs2472297))	No	No
*Articles evaluating at least two of the outcomes (CHD, MI, CVD or Stroke)*									
Livingstone M et al. [77]	United Kingdom (not described)	Cohort (Population)	UK Biobank (7.8)	1141 MI, 748 IS/77004	Both	Recommended Food Score RFS(Oxford WebQ (24-h dietary assessment tool))	-(GRS formed from over 300 different SNPs associated with CVD)	Yes	No
Hellstrand S et al. [76]	Sweden (White)	Cohort (Population)	MDCS (15)	3068/24799	Both	Diet quality index (168-item dietary questionnaire 7-day menu book 1-h diet history interview)	-(Genetic risk score for LDL, HDL and Triglycerides)	Yes	No
Hellstrand S et al. [6]	Sweden (not described)	Cohort (Population)	MDCS (14)	2648/24032	Both	PUFAs (Questionnaire)	FADS1 (rs174546)	Yes	No
Zee R et al. [79]	United States (White)	Cohort (Population)	WHS (9.9)	812/24968	Women	Folate intake, VB2, VB6 and VB12 intake (Questionnaire)	MTHFR (677C > T)	No	No
Heianza Y et al. [75]	United Kingdom (not described)	Cohort (Population)	UK Biobank (5)	1812/156148	Both	Plant-based diet index (Participants completed a web-based 24-h dietary assessment, the Oxford WebQ, during 2009–2012.)	Genetic risk score GRS for stroke and myocardial infarction ()	No	No
Do R et al. [17]	Multicentre (European, South Asian, Chinese, Latin American, Arab)	Case–control/ cohort (Population)	INTERHEART study and FINRISK study (NR)	3709/27243	Both	Dietary risk score and specifically different types of food (FFQ)	9p21 locus (rs10757274, rs2383206, rs10757278, rs1333049)	Yes	No
Miao L et al. [78]	China (Han-Chinese)	Case–Control (Clinical)	–	846/2562	Both	Alcohol (Questionnaire)	MVK-MMAB (rs3759387, rs7134594, rs877710, rs9593)	Yes	No
Corella D et al. [74]	Spain (Mediterranean)	Randomised controlled trial (Clinical)	PREDIMED Trial (4.8)	268/7187	Both	Mediterranean diet, extra-virgin olive oil + nuts(NR)	LPL (rs13702)	Yes	No
Bergholdt H et al. [72]	Denmark (European descent)	cross-sectional and Mendelian randomization (Population)	CCHS, CGPS and GESUS (5.4)	10,372 IHD, 4188 MI /98529	Both	Milk (Questionnaire)	LCT13910 (rs4988235)	No	No
Liu CX et al. [81]	China (Han-Chinese)	Case–Control (Clinical)	–	622/1260 CAD, 593/1231 stroke	Both	Alcohol(NR)	EHBP1, TUBB, and WWOX (rs2710642, rs10496099, rs3132584, rs3130685, rs2222896, and rs2278075)	Yes	No
Zheng PF et al. [80]	China (Han-Chinese)	Case–Control (Clinical)	–	758/1513 CAD, 756/1511 stroke	Both	Alcohol (NR)	SYTL3 (rs9364496, rs6455600, rs2129209 and rs9456350) and SLC22A3 (rs446809 and rs539298)	Yes	No
Corella D et al. [73]	Spain (Mediterranean)	Randomised controlled trial (Clinical)	PREDIMED Trial (4.8)	150/7098	Both	Mediterranean diet supplemented with extra virgin olive oil (Validated FFQ)	CLOCK (rs4580704)	No	No

*FU* Follow-up; *IS* Ischemic stroke; *MI* Myocardial infarction; *CHD* Coronary heart disease; *CVD* Cardiovascular diseases. *NR* Not reported; *BWHHS* British Women's Heart & Health Study; *CCHS* Copenhagen City Heart Study; *EPIC* European prospective investigation into cancer and nutrition cohort; *FISSIC* Fangshan/family-based ischemic stroke study in China; *KIHD* Kuopio ischaemic heart disease risk factor study; *NPHS II* Second Northwick park heart study; *TWB* Taiwan Biobank; *WHS* Women's Health Study

There were 52 genetic factors (GRS, genes, SNPs) and 50 different dietary exposures studied. A description of the dietary scores, indexes, or patterns reported can be found in Additional file 2: Table S2. The most investigated dietary component was alcohol, studied in 30 (50.8%) articles, and ADH1C studied in 7 (11.9%) articles. Regarding genetic information, 29 (49.2%) articles did not present MAF (Additional file 2: Table S2). Regarding outcome measurement, 28 (47.5%) articles included prevalent CVD cases, and 31 (52.5%) articles included the incidence of CVD cases. Overall, the median CVD events was 759, ranging from 72 to 10,372. Four (6.8%) articles replicated their findings in different samples (Table 1).

#### Characteristics of included articles reporting significant gene-diet interactions

In total, 31 articles reported significant gene-diet interactions. Among the articles reporting significant interactions, the most frequent place of publication was China with 13 (41.9%) articles, followed by Europe with ten (32.3%) and Latin America with five (16.1%). The case–control design was reported in 22 (71%) articles; the median sample size was 3311, ranging from 200 to 77,004. Four (13%) articles evaluated the interaction between alcohol and the cholesterol ester transfer protein (CETP) rs708272 variant, being this interaction the most frequently evaluated.

### Gene-diet interactions and coronary heart disease.

**Fig. 2:**
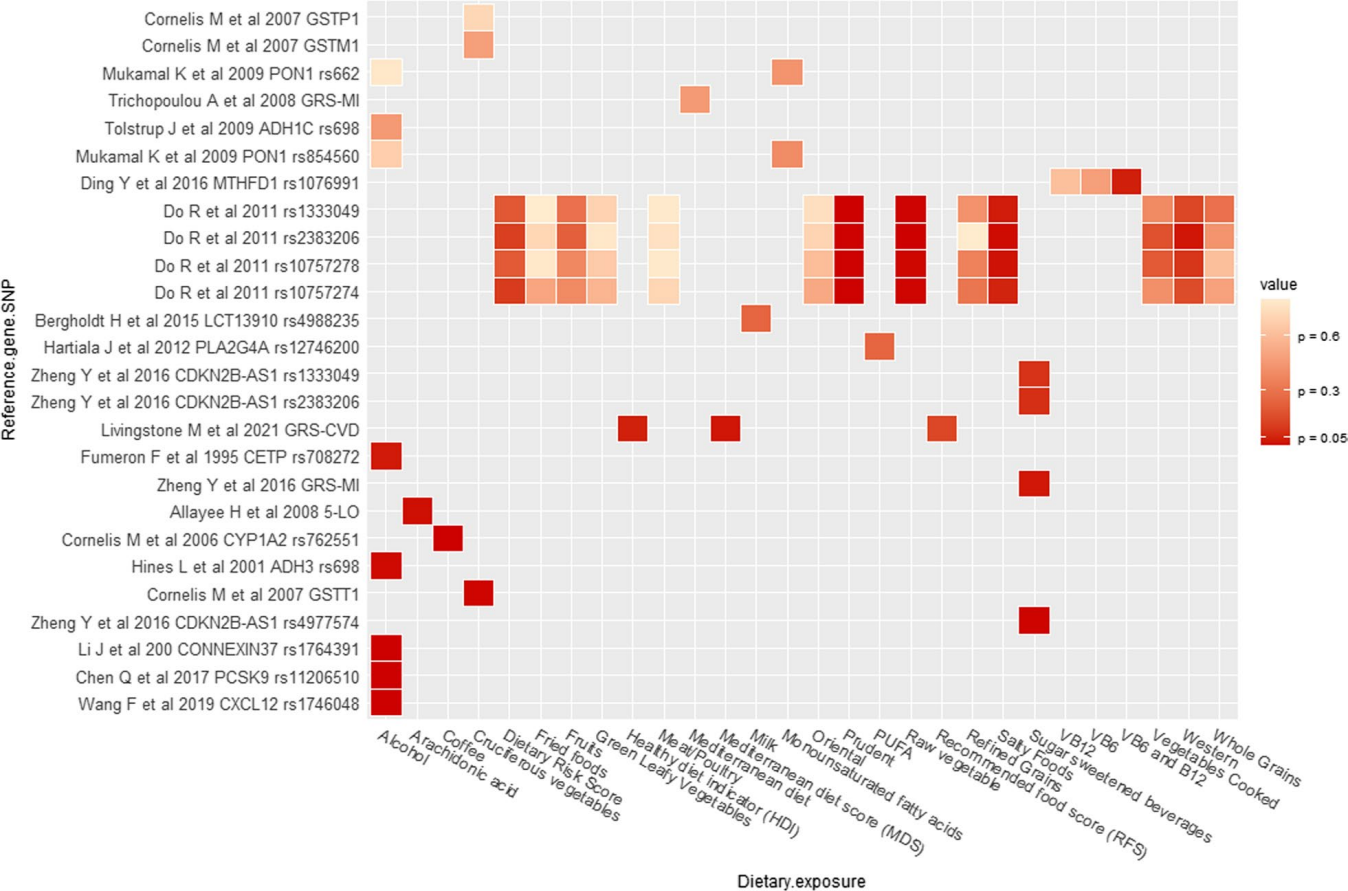
Findings for interaction between genetic variants and diet in relation to myocardial infarction. W = women, M = men, B = Both (Men and women)

**Fig. 3:**
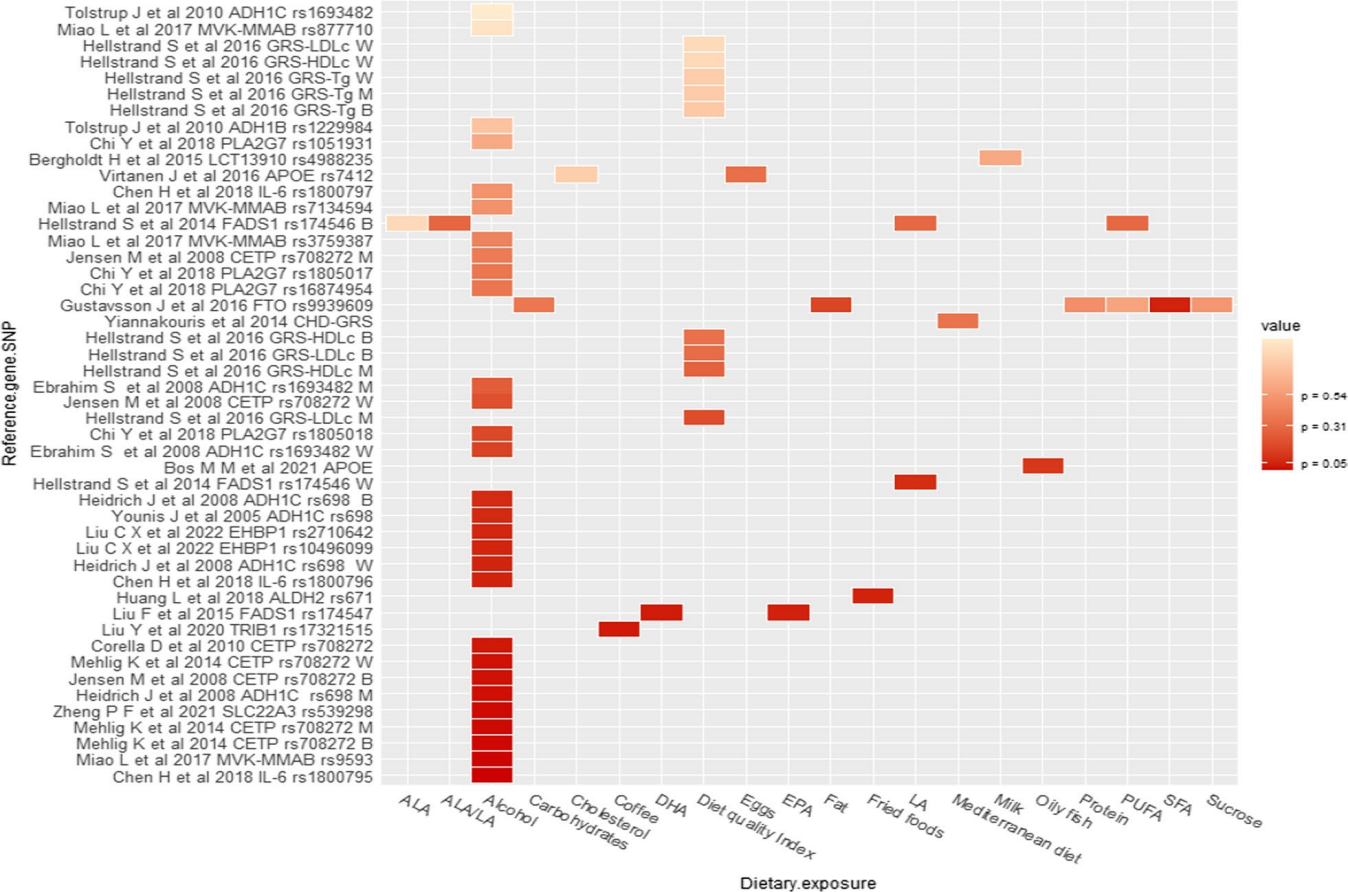
Findings for interaction between genetic variants and diet in relation to coronary heart diseases. W = women, M = men, B = Both (Men and women)

Thirty three articles from 21 unique studies evaluated whether specific nutrients, foods or diets modified the association between genetic factors and CHD (Figs. 2 and 3, Table 1) [11–13, 16, 31–57, 80, 81]. The most frequently evaluated dietary exposure and genetic variants were alcohol (n = 17) and *ADH1C* (n = 6), respectively. *CETP* TaqIB was the second most evaluated genetic variant; estimations for alcohol-*ADH1C and -CETP* interactions on CHD risk can be found in Table 2. The main findings regarding non-significant interactions in the macronutrients category were that PUFA intake did not interact with PLA2G4C, FADS1 or FTO variants on CHD risk. Micronutrients such as folate and vitamin B did not interact with the *MTHFR* 677CT variant. Other non-significant interactions were milk-LCT-13910, fried food-*ALDH2*, (dietary) cholesterol-*APOE*, alcohol-*ADH1C*, -*CETP*, -*PON1*, -*PLAG2G7*, -*TFPI-2*. Similarly, dietary scores did not significantly interact with GRS of HDL, LDL, triglycerides, or MI [6, 11–13, 17, 36, 40, 44–48, 52–57, 72, 76, 79, 81]. An overview of the non-significant interactions can be found in Figs. 2 and 3, and more details are provided in Additional file 2: Table S3. In the following paragraphs, we will discuss the findings of the articles that reported significant interactions.

**Table 2:** Estimates of the interaction between alcohol intake and ADH1C variants on CHD risk

Author	Categorization of Alcohol (Drinks/week)	No. of Events	Association measure	Gene, variant, and genotypes Estimate (CI 95%)			Interaction *P-*value
				CETP (rs708272 (CETP/TaqIB))			
				B1B1	B1B2	B2B2	
*CHD*							
*Fumeron et al. [35]	Non-drinkers	92	OR	1	1.04 (0.68–1.59)		<0.02
	<2	234		1	0.97 (0.58–1.61)		
	≥2 to 3	134		1	0.96 (0.51–1.81)		
	≥4 to 5	66		1	0.56 (0.22–1.47)		
	≥6	125		1	0.34 (0.14–0.83)		
Jensen et al. [12]	Non-drinkers^a^	118	OR	1	1		0.4
	<2.5^a^	77		1.1 (0.5–2.3)	0.8 (0.5–1.4)		
	≥ 2.5 to 6^a^	31		1.4 (0.6–3.7)	0.3 (0.2–0.6)		
	≥ 7 to 14^a^	20		1.3 (0.5–3.8)	0.4 (0.2–0.9)		
	Non-drinkers^b^	63		1	1		0.2
	<2.5 ^b^	63		1.7 (0.7–4.1)	0.9 (0.5–1.6)		
	≥ 2.5 to 6^b^	66		1.9 (0.8–4.5)	0.9 (0.5–1.6)		
	≥ 7 to 14^b^	80		1.6 (0.6–4.4)	0.8 (0.4–1.5)		
	Non-drinkers^c^	181		1	No data		
	≥ 2.5 to 6^c^	87		1.6 (1.1–2.3)	0.7 (0.6–1.0)		0.02
Corella et al. [45]	Non-drinkers	139	OR	1	0.74 (0.42–1.32)	0.57 (0.24–1.34)	0.031
	Drinkers	418		1	1.17 (0.90–1.55)	1.55 (1.05–2.29)	
Mehlig et al. [51]	Abstainers			1.12 (0.77–1.62)		0.76 (0.36–1.64)	0.008
	Low			1		1	
	Intermediate			0.80 (0.59–1.06)		0.21 (0.10–0.44)	
	High			1.03 (0.77–1.36)		0.48 (0.26–0.88)	

Author	Categorization of Alcohol (Drinks/week)	No. of Events	Association measure	Gene, variant, and genotypes Estimate (CI 95%)			Interaction *P-*value
				ADH1C			
				1/1	1/2	2/2	
*Tolstrup et al. [39]	<1	175	HR	1	1.38 (0.97–1.96)	1.60 (1.04–2.47)	0.49
	1 to 13	307		0.99 (0.70–1.40)	0.98 (0.71–1.37)	0.83 (0.55–1.25)	
	≥14	146		0.80 (0.53 – 1.23)	0.82 (0.56–1.19)	0.88 (0.55–1.42)	
*Heidrich et al. [47]	<1	24	HR	1	0.69 (0.31–1.55)		0.07
	1 to 6	13		0.56 (0.19–1.61)	0.83 (0.34–2.07)		
	≥7	35		1.06 (0.50–2.25)	0.36 (0.16–0.80)		
*Younis et al. [55]	<1	44	HR	1	0.82 (0.47–1.45)	0.64 (0.24–1.68)	0.49
	1 to 6	64		0.70 (0.40–1.22)	0.56 (0.32–0.99)	0.66 (0.31–1.38)	
	≥7	102		0.57 (0.33 – 0.98)	0.77 (0.47–1.26)	0.68 (0.36–1.27)	
*Hines et al. [37]	<1	117	RR	1	1.01 (0.58–1.75)	0.59 (0.28–1.23)	0.01
	1 to 6	191		1.11 (0.67–1.84)	0.66 (0.40–1.08)	1.02 (0.55–1.88)	
	≥7	87		0.62 (0.34 – 1.13)	0.68 (0.40–1.15)	0.14 (0.04–0.45)	
Tolstrup et al. [13]	<1	68	HR	0.96 (0.47–1.93)	1.86 (0.94–3.65)	1.45 (0.47–4.47)	0.95
	1–6	230		1	1.38 (0.87–2.19)	1.10 (0.59–2.08)	
	7–20	266		0.88 (0.56–1.39)	0.97 (0.62–1.51)	0.91 (0.52–1.58)	
	>21	206		0.97 (0.59–1.59)	0.73 (0.45–1.19)	0.84 (0.46–1.54)	
*Ebrahim S. et al. [11]	No data						0.26
*CVD*							
Djoussé et al. [69]	0	56	OR	1	0.85 (0.43–1.68)	1.10 (0.47–2.54)	0.48
	>0	76		0.90 (0.49–1.67)	0.72 (0.39–1.31)	0.63 (0.28–1.44)	

1 = Reference category. *Articles reporting grams/day were transformed into drink/week taking as reference "standard" drink (or one alcoholic drink equivalent) contains roughly 14 g of pure alcohol [30]

^a^ Women estimates (Nursing Health Study data), ^b^ Men estimates (Health Professional Study HPFS), ^c^ estimates from a pooled dataset (NHS + HPFS)

*HR*  Hazar ratio; *RR* Relative risk; *OR* Odds ratio

Regarding macronutrients, in a Costa Rican case–control study including approximately 3800 patients, Allayee et al. [31] reported a significant (*p* = 0.015) interaction between arachidonic acid (AA) and 5-lipoxygenase (*5-LO*) promoter variants [31]. Consumers of ≥ 0.25 g/day of AA who carried one or two copies of the shorter three and four repeats of *5-LO* had a higher MI odds ratio (OR) 1.31 (95% CI 1.07, 1.61) than consumers of < 0.25 g/day of AA who are 55 homozygote carriers. In comparison, among consumers of < 0.25 g/day of AA who were carriers of one or two copies of the shorter three and four repeats, lower odds was observed [OR 0.77 (95% CI 0.63, 0.94)] [31]. In the same study, Hartiala et al. found a significant (*p* = 0.005) interaction between PUFA and a variant of *PLA2G4C* (rs12746200) [36]. Subjects with high dietary n-6 PUFA intake (≥ 6.93 g/day) who were carriers of AG/GG genotype had lower odds for MI [OR 0.71 (95% CI 0.59, 0.87)] than AA homozygote subjects [36].

In a case–control study using Wuhan (China) data, Liu F et al. [49] found a significant (*p* = 0.028) interaction between PUFA and a variant of *FADS1* (rs174547). Subjects in the lowest tertile of EPA and DHA intake who are carriers of T alleles had higher odds of developing CHD [OR 3.04 (95% CI 1.94, 4.76)] and [OR 2.56 (95% CI 1.64, 3.98)], respectively, compared to subjects in the highest tertile of EPA intake and DHA consumption, who are also carriers of rs174547 C/C genotype. No association was observed in the middle tertile of EPA or DHA intake [49].

Regarding micronutrients, the Western Norway B-vitamin intervention randomised trial (WENBIT) prospectively evaluated interactions between folic acid, vitamins B12/B6 and an *MTHFD1* variant (rs1076991) in 2381 participants [16]. In this trial, carriers of the rs1076991 T allele who received folic acid/vitamin B12 and vitamin B6 combined treatment had a hazard ratio (HR) for MI of 2.35 (95% CI 1.55, 3.57) (*p* = 0.047) when compared to the placebo group. On the other hand, no association with MI was observed in the groups who had vitamin B6 or folic acid/B12 separately [16].

In the food and food items categories, a case–control study using data from 52 countries (the INTERHEART study) [17], and a case–control study analysing data from a Hispanic population [33], reported interactions between high vegetable intake and four variants (rs10757274, rs2383206, rs10757278, rs1333049) of the chromosome 9p21 [17] and the Glutathione S-transferase theta 1 (*GSTT1*) gene variants [33]. Subjects whose vegetable intake was classified in the highest tertile who were carriers of the functional GSTT1*1 allele had lower odds for MI [OR 0.70 (95% CI 0.58, 0.84)] compared to those whose intake was classified in the lowest tertile (*p* = 0.006) [33]. In contrast, carriers of risk alleles of 9p21 variants had a lower incidence of MI among participants who consumed vegetables daily (*p* < 0.008) [17]. However, the interaction with 9p21 variants was not significant when restricted to cooked vegetables [17].

In a case–control study using data from the same Hispanic population mentioned above, Cornelis et al. reported a significant (*p* = 0.04) interaction between coffee consumption and *CYP1A2* variants on MI risk [34]. The consumers of ≥ 4 cups/day of coffee carrying the rs762551 variant had higher odds of MI [OR 1.64 (95% CI 1.1, 2.34)] [34] compared to those consumed < 1 cup/day. Conversely, a study from Taiwan Biobank (TWB) found a significant (*p* = 0.03) interaction between coffee consumption and a tribbles pseudokinase 1 (*TRIB1*) variant rs17321515 on CHD. Those who drank coffee and were carriers of the GG genotype had reduced odds of CHD [OR 0.62 (95% CI 0.45, 0.85)] compared with non-coffee drinkers [50].

Concerning other dietary components, in a case–control study from the *Etude Cas-Témoin de l'Infarctus du Myocarde* (ECTIM) (n = 724), alcohol consumption significantly (*p* < 0.005) interacted with the *CETP* TaqIB variant (rs708272). Subjects who consumed 50 g/day or more alcohol and were TaqIB B2B2 homozygotes had a lower odds of MI [OR 0.39 (95% CI 0.20, 0.75)] compared with those who consumed < 50 g/day. Additional analysis comparing different alcohol intake categories through B2B2 heterozygotes with B1B1 and B1B2 genotypes found that the protective effect of B2/B2 genotype was significant (*p* < 0.02) in the category of ≥ 6 drinks per week, Table 2 [35]. Three more authors reported interaction between alcohol and the same variant [12, 45, 51]. Jensen et al. [12] reported a significant interaction (*p* = 0.02) among drinkers of 5–14.9 g/day of alcohol who were B2 carriers, who had a lower odds of MI [OR 0.7 (95% CI 0.6, 1.0), compared with non-drinkers, however, no significance was observed when the analysis was stratified by sex [12]. Similarly, Mehlig et al. [51] reported that subjects classified in the second [OR 0.21 (95% CI 0.10, 0.44)] and third tertile [OR 0.48 (95% CI 0.26, 0.88)] of alcohol intake who were B2/B2 homozygotes had lower MI odds (*p* = 0.008), compared with those in the first alcohol intake tertile. When the analysis was performed by sex, significance was only reported in men [51]. Conversely, Corella et al. [45], evaluating the effect of alcohol consumption and the TaqIB variant, found that B2/B2 homozygotes had an increased odds of CHD [OR 1.55 (95% CI 1.05, 2.29), *p* = 0.031], compared with B1B1 genotype [45], Table 2.

Similarly, a nested case–control study from the Physicians' Health Study (n = 1166) reported a significant (*p* = 0.01) interaction on MI risk between alcohol consumption and ADH1C. The lowest risk was observed in those who consumed ≥ 1 drink per day and carried ADH1C (^γ2^ ^γ2^), compared with those who consumed ˂1 drinks per week [RR 0.14 (95% CI 0.04, 0.45)] [37]. Other studies evaluated the interaction between alcohol and ADH1C but reported no significant interactions (Table 2 and Additional file 2: S3).

Han Chinese population matched case–control studies found increased risks of MI due to the interaction of alcohol consumption with the *CXCL12* rs1746048 and *PCSK9* rs11206510 variants [32, 41] (*p* < 0.001). Participants with the rs1746048 CC genotype and rs11206510 TT genotype consuming 0–250 g/day of alcohol had an MI OR of 14 (95% CI 3.2, 61.4) and 9.63 (95% CI 3.7, 24.9), respectively [32, 41], compared to non-drinkers. By contrast, within the same categories of alcohol intake, carriers of the *Cx37* variant rs1764391 with CC genotype had an OR 0.54 (95% CI 0.31, 0.9) [38]. An increased odds of MI was observed between those consuming ≥ 250 g/day alcohol who carried the rs1764391 CC genotype, rs1746048 CC genotype, and rs11206510 TT genotype, with ORs of 32.7 (95% CI 4.4, 241.6), 24.0 (95% CI 4.9, 116.3), and 14.0 (95% CI 5.1, 42.1), respectively [32, 38, 41]. Additionally, in the same population, carriers of the SLC22A3 variant rs539298 with AG/GG genotype who reported alcohol drinking had an OR 0.53 (95% CI 0.37, 0.77), compared with no drinkers [80].

A case–control study by Zheng et al. [42] analysed data from a Hispanic population and reported a significant (p = 0.03) interaction between SSB consumption and the GRS of 9p21 variants (rs4977574, rs2383206, rs1333049). The OR of an MI incident (per allele risk of GRS) was 1.00 (95% CI 0.94, 1.07) in participants with SSB intake of < 1 serving/day, 1.07 (95% CI 0.99, 1.14) in participants with an intake of 1–2 servings/day, and 1.12 (95% CI 1.05, 1.20) in participants with an intake of  >  2servings/day [42]. Additionally, a case–control study from the Nanning province (China) showed that participants who consumed alcohol and were carriers of the mevalonate kinase *(MVK)* variant rs3759387 with AA/AC genotypes had reduced odds of having CHD [OR 0.66 (95% CI 0.38, 1.03, *p* < 0.001], compared to non-drinkers [78]. On the contrary, a study performed in Wuhan (China) found a significant (*p* = 0.001) interaction between alcohol intake and Interleukin-6 (*IL-6*) variant rs1800795; current drinkers who were carriers of the rs1800795-C allele had an OR of 3.17 (95% CI 2.20, 4.24) [43], compared to never-drinkers.

In terms of dietary scores/indices, in a prospective analysis comprising 77,004 participants from the UK Biobank, Livingstone et al. [77] reported a marginal (*p* = 0.049) interaction between Healthy Diet Indicator (HDI) (Additional file 2: Table S2) and GRS-CVD. In addition, the study found a significant (*p* = 0.026) interaction with the MDS and GRS-CVD on the risk of MI (Additional file 2: Table S2); individuals adhering to the Mediterranean diet (high MDS) with higher genetic CVD risk had a stronger risk reduction [HR 0.91 (95% CI 0.85, 0.97)]. In comparison, there was no evidence of an interaction of MDS on MI in participants with low GRS-CVD [HR 1.03 (95% CI 0.94, 1.12)] [77].

### Gene-diet interactions and stroke

**Fig. 4:**
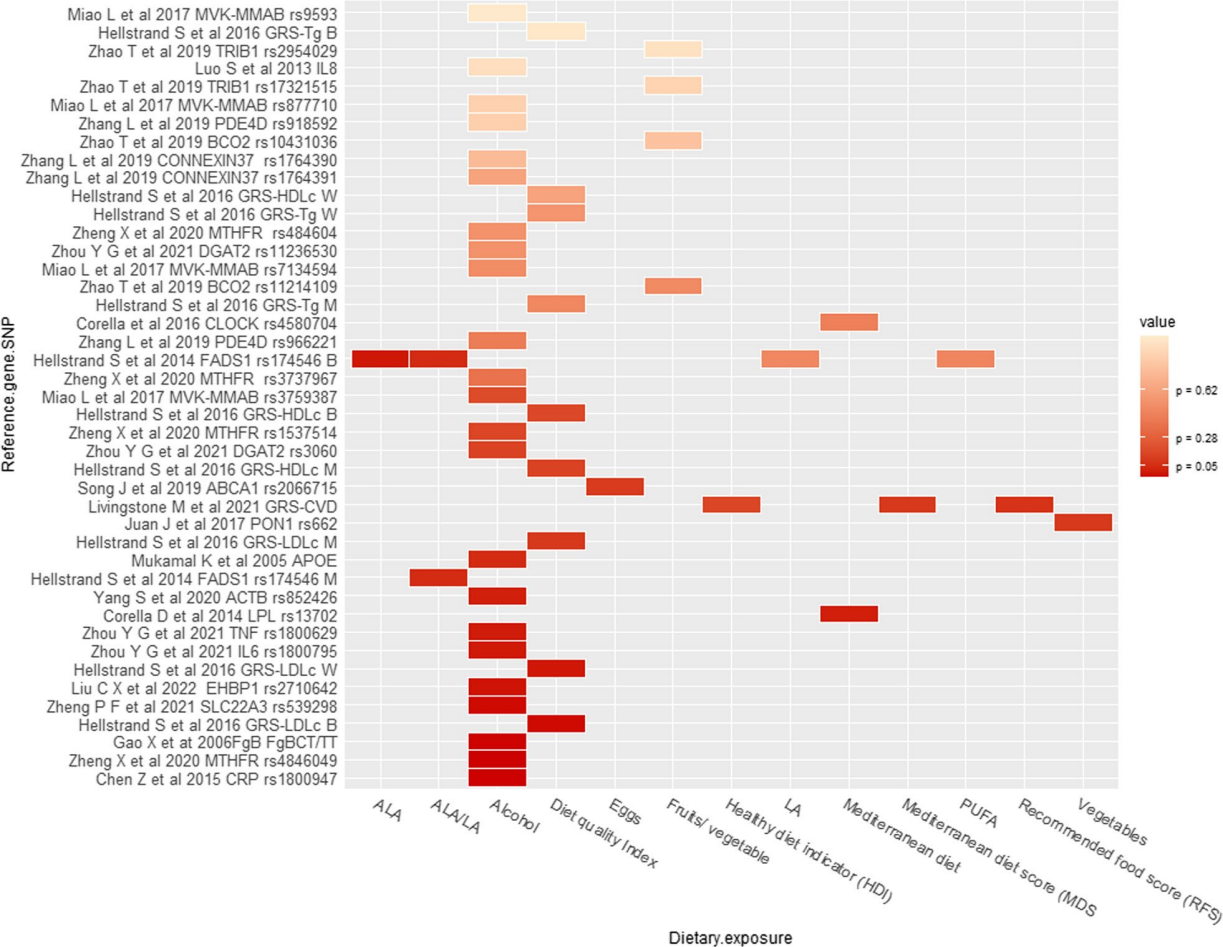
Findings for interaction between genetic variants and diet in relation to stroke. W = women, M = men, B = Both (Men and women)

Twenty two articles from 14 unique studies evaluated whether specific foods or diets modified the association between genetic factors and stroke (Fig. 4, Table 1) [6, 14, 58–66, 73–79]. Non-significant interactions were reported for alcohol intake and *APOE, IL-8* variant, *PDE4D*, DGAT2, *CONNEXIN37* genes. Similarly, different dietary scores did not interact with *CLOCK* gene variants or GRS-CVD and GRS-stroke [61, 64–66, 68, 73, 76, 78], Additional file 2: Table S3.

In the macronutrients category, the MDC cohort study evaluated interactions between fatty acids and the *FADS1* rs174546 variant. This study found that only the interaction between ALA and FADS1 rs174546 TT genotype was significant (p = 0.03). Participants in the higher ALA consumption quintile carriers of TT genotype had a decreased risk of stroke [HR 0.50 (95% CI 0.27, 0.94)], compared to carriers of the TT genotype in the lowest quintile of ALA intake. At the same time, no association was observed in CC and CT genotypes in the other quintiles [6].

Within the food and food items categories, the FISSIC found a significant (*p* = 0.006) interaction between the egg intake and ABCA1 variant (rs2066715) [63]. In the same study, a significant interaction between vegetable intake and the *PON1* rs662 variant on the risk of stroke was found. Each standard deviation increment in vegetable intake was associated with a 40% reduction in the risk of stroke among carriers of the *PON1* rs662 AA genotype. On the contrary, each standard deviation increment in vegetable intake was associated with a 51% increased risk of stroke among rs662 GG carriers; after adjustment for fruit intake, the interaction was not significant (*p* = 0.12) [60].

Concerning other dietary components, a case–control study from Beijing in China found a significant (*p* = 0.001) interaction between alcohol and *CRP* variant rs3093059. Drinkers with the rs1800947 GC [OR 11.11 (95% CI 1.22, 100.45)] and GG genotypes [OR 2.99 (95% CI 1.73, 5.19)] had an increased odds of having a stroke compared with non-drinkers and carriers of GG genotype. On the other hand, non-drinkers with the rs1800947 GC genotype had an OR of 2.95 (95% CI 1.05, 8.29) [58]. Similarly, another case–control study in a Chinese Han population found a significant (*p* = 0.003) interaction between drinking status and the *Fgβ* 148CT variant. Drinkers who are also carriers of CT/TT genotype had increased odds of having a stroke (OR 22.7 (95% CI 2.95, 173.76) compared to non-drinker carriers of the CC genotype [59]. Another case–control study from the Community Hypertension Survey in the Chinese city of Yixing found a significant (*p* = 0.048) interaction between drinking status and rs852426 β-actin (ACTB) variant on stroke risk [HR 0.54 (95% CI 0.29, 0.99)] [14]. Another Han population case–control study found a significant (*p* = 0.001) interaction between alcohol status and rs4846049. Drinkers with rs4846049 CA/AA genotype had an OR of having a stroke of 3.12 (95% CI 1.83, 4.45) compared with never drinkers and rs4846049 CC genotype. None of the other *MTHFR* variants evaluated significantly interacted with alcohol [66].

In the category of dietary patterns, the PREDIMED trial found a significant (*p* = 0.04) interaction between the Mediterranean diet and the LPL rs13702 variant. Participants assigned to the intervention group (Mediterranean diet plus supplementation with extra-virgin olive oil and nuts (30 g/day)) who were carriers of the C allele had a reduced stroke risk [HR 0.58 (95% CI 0.37, 0.91)] in comparison to the TT genotype. At the same time, no association was reported for the control group (fat intake reduction) [74]. Finally, Helstrand et al. [76], analysing data from the MDC cohort, reported a significant (*p* = 0.04) interaction between diet quality index and GRS-LDL-cholesterol on stroke risk (Additional file 2: Table S2). Participants with low/medium diet quality had a HR of 1.09 (95% CI 1.03, 1.16) per standard deviation of increment of GRS-LDL-cholesterol [76].

### Diet-Gene interactions and cardiovascular diseases as a composite outcome

**Fig. 5:**
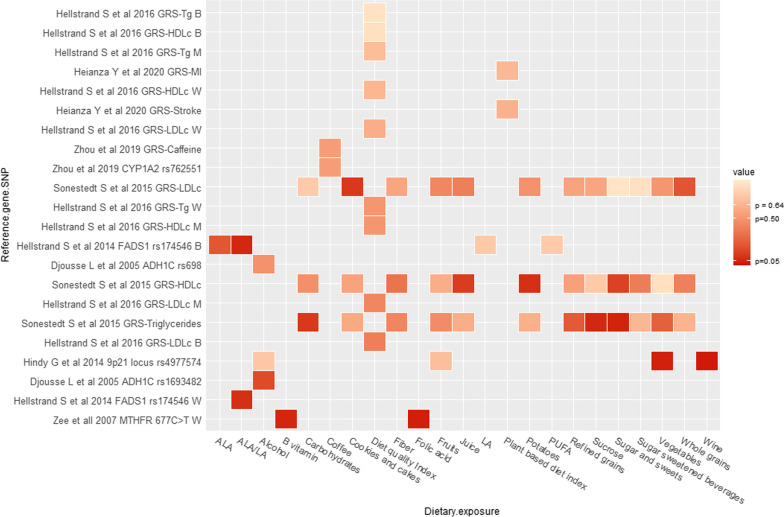
Findings for interaction between genetic variants and diet in relation to cardiovascular diseases as composite outcomes. W = women, M = men, B = Both (Men and women)

Eight articles from four unique studies evaluated diet-gene interactions on cardiovascular diseases as composite outcome [6, 15, 56, 69, 70, 75, 76, 79] (Fig. 5, Table 1). Non-significant interactions were reported for drinking status-ADH1C variant, and diet quality with GRS of HDL, -LDL and -triglycerides [6, 69–71, 75, 76], Additional file 2: Table S3.

In the macronutrients category, a borderline (*p* = 0.06) interaction was reported between ALA/LA intake ratio and the *FADS1* variant on CVD incidence. No statistically significant interaction was observed with any of the other fatty acids evaluated [6]. Regarding micronutrients, neither folate nor vitamin B intake interacted with MTHFR variants on CVD risk [79].

Regarding food and food items categories, Hindy et al. [70], analysing data from the MDC cohort, reported a significant (*p* = 0.043) interaction between vegetable intake and chromosome 9p21 variant rs4977574. When the analysis was restricted to medium or high tertile of vegetable intake, carriers of the G allele had an increased risk of CVD with HR 1.27 (95% CI 1.17, 1.38) and 1.19 (95% CI 1.08, 1.30), respectively, compared to AA homozygote genotype. No interaction was reported for fruit intake [70]. Moreover, Sonestedt et al. [15], in another analysis of the same MDC cohort, found no interaction between vegetable intake and GRS of HDL cholesterol, LDL cholesterol or triglycerides on CVD risk [15]. Additionally, in the UK Biobank, there was no interaction between coffee intake and CYP1A2 genotype or with a GRS of caffeine metabolism on CVD risk (*p* > 0.53) [71].

Concerning other dietary components, in the MDC cohort, a significant (*p* = 0.029) interaction was found between wine consumption and chromosome 9p21 variant rs4977574 on CVD risk. However, the effect was limited to the non/low wine intake tertile in the stratified analysis. In that group, carriers of the G allele had an increased risk of CVD [HR 1.23 (95% CI 1.14, 1.34)] compared to the AA homozygote genotype. At the same time, no association was observed when total alcohol intake was evaluated [70].

#### Risk of bias of the included studies

Twenty one (35.6%) articles were classified as high quality, 36 (61.0%) as intermediate quality, and two (3.3%) as poor quality. Small sample size, lack of correction for multiple testing (11 (18.6%) articles adjusted for multiple comparisons), lack of generalisation (e.g., no different ethnicities being represented) often limited the methodological quality (Additional file 2: Table S4), a report of the SWiM items can be found in Additional file 2: Table S5.

## Discussion

Of the 59 included articles, 32 reported a statistically significant gene-diet interaction. Dietary and genetic exposure were very heterogeneous, which precluded us from conducting a meta-analysis of the results. *CETP* and alcohol dehydrogenase (*ADH1C*) variants were the most frequently assessed and were shown to interact with alcohol to modify the risk of MI and CHD. Other studies investigating plausible biological interactions such as *FADS* gene and fatty acids interactions, vitamin B6, vitamin B12 and folic acid did not show consistent findings. While several studies investigated the interactions between genes and dietary factors on CVD risk, the current literature is limited and not consistent in showing gene-diet interactions with clinical and public health impacts, mainly because the reported positive findings were derived from case–control studies and lacked replication.

Previous systematic reviews on gene-diet interactions and CVD have primarily focused on specific genes or diets. In contrast, our study provides a comprehensive assessment of all genes and dietary exposures interactions on CVD. Similar to previous findings, we identified a lack of consistency in the results of interaction studies [19, 82]. In this review, the lack of reproducibility in the genetic-dietary variables operationalisation and the different levels of validation and reliability of the used dietary questionnaires could have led to an increased risk of exposure misclassification. This risk could be more relevant in case–control studies, in which recall bias could occur differently between cases and controls since the cases are aware of the condition [83]. Additionally, misclassification due to genotype errors can be another source of bias. Genotyping error has been reported to vary between about 1% and 30%, and its extension is related to variations in DNA sequence, quality of the analysed DNA, biochemical artefacts and human factors [84].

Another methodological concern of studies looking at gene-diet interaction and CVD is the sample size of the studies. Low statistical power leads to a reduced capacity to detect interactions. Genotyping errors, allele frequency and the precision of the dietary exposure and outcome measures are some of the criteria that researchers should consider when calculating adequate sample size to evaluate interactions [85]. Nevertheless, most of the studies included in this paper were secondary analyses, and there was no information on whether studies had enough power to detect an interaction. It has been estimated that detecting the interaction between two binary exposures requires a sample size four times larger than that required to detect main associations of the same magnitude [86].

Similarly, studies with 95% of power and a MAF of 20% looking for interactions of 1.5 of magnitude between genetic variants and continuous exposures require a sample size of up to 30,906 subjects [86]. In this paper, 50% of the included studies had a sample size below 2562 individuals. Just four studies exceeded 30,000 participants, and two of them did not clearly state the MAF frequency [75, 77]. The lack of information on the main factors involved in calculating power in almost half of the included studies limited the evaluation of their sample robustness for detecting gene-diet interactions. Notably, of the four studies that exceeded 30,000 participants, only one found a significant interaction [77].

Comparing specific foods and gene variants generates multiple comparison scenarios that could increase the Family-wise error rate [87], where the probability of false-positive findings increases with each additional comparison [88]. Therefore, including a correction for multiple testing is a suitable approach in studies with these phenomena, even though in this study, just two studies stated a correction for multiple comparisons in their methodology [17, 78].

Alcohol was the most evaluated exposure; its interaction with the CETP polymorphism (rs708272) was not consistent for CHD. The results did not agree with the direction of reported interactions, and most of the interactions lost statistical significance in the sex-stratified analysis. The low prevalence of alcohol intake could explain this difference and hypertriglyceridemia in the populations evaluated. [12, 45]. In addition, only two studies included incident cases. However, the protective effect of the CETP-alcohol interaction could be related to the synergy between the B2 allele of CETP, which is associated with lower plasma CETP activity [89], and the inhibitory effect of alcohol on CETP activity [12]. Both may increase HDL concentrations, decrease LDL and VLDL fractions, and, consequently, reduce CVD risk.

Similarly, concerning lipid metabolism, a matched case–control study reported an interaction between the *ADH1C* variant and alcohol intake that decreases the incidence of MI in men who drank daily and were homozygous for the γ2 allele. Carriers of the γ2 allele are slow metabolisers of alcohol, which could enhance the beneficial effect of moderate alcohol consumption on lipid metabolism. In addition, the study stated that up to 50% of the observed decrease in MI risk could be attributed to increased HDL levels [37]. However, findings on *ADH1C* polymorphism and alcohol interactions were not homogeneous, and five studies did not report significant interactions, even though different alcohol intake categories were tested among these studies [11, 13, 39, 47, 55]. These findings suggest that the interactions between alcohol consumption and the *ADH1C* variant on CVD might be mediated through mechanisms independent of HDL cholesterol [69].

The increased risk of MI in the WENBIT trial could be explained by the association of vitamin B6 and folate intake with elevated hepatic adenosylmethionine (SAM). SAM is an inhibitor of betaine-homocysteine methyltransferase, an enzyme that regulates hepatic lipids and induces ApoB expression and VLDL assembly. Furthermore, the *MTHFD1* variant (rs1076991) minor T-allele has been associated with an approximately 62.5% drop-in transcription rate of the MTHFD1 enzyme, which could also be associated with intercellular SAM accumulation, conditions that lead to dyslipidaemia and the consequent increased CVD risk [16]. However, when MI was evaluated as part of CVD composite outcome or individually in WHS, the folate or B-vitamin—MTHFD1 interaction was not found [79]. It is important to note that meta-analyses of the association of MTHFR and CVD have found substantial geographical heterogeneity and null associations for MTHFR and CVD in North American populations, such as women involved in the Women's Health Study [79].

### Strengths and limitations

A significant strength of this paper is the comprehensive search strategy implemented to retrieve gene-diet interaction studies. We included all food and dietary exposures and epidemiological designs, providing a comprehensive overview of the literature. Also, we provided a critical evaluation of the quality of the current evidence on the topic. In addition, the included studies point to several biological mechanisms that could underlie the differences in the susceptibility to food/diet exposures and cardiometabolic diseases. However, it is a limitation for this study that, so far, no gene-diet interaction critical appraisal tool has been developed. This tool could standardise the evaluation of the studies' risk of bias and methodological quality, identifying the most significant weaknesses. Other issues were the lack of replication in the evaluation of interactions, few studies evaluated the same dietary and genetic exposures (SNP, GRS). Moreover, authors evaluating the same genetic variants used different genetics models (e.g. recessive model, co-dominant model or dominant model). This heterogeneity limited the synthesis of the findings and are also a great weakness for the progress in the identification of population at higher risk of cardiometabolic diseases due to their genetic background and food/diet exposures.

### Future research and implications

Identifying the mechanisms underlying gene-diet interactions is a priority; therefore, variants identified in GWAS are required to be investigated in functional studies, a challenge that could benefit from computational modelling. In addition, studies assessing interactions should provide more information on the origin of biases in the genetic exposures assessed (genotype misclassification, population stratification). Future studies should analyse samples with a suitable size for evaluating interaction hypotheses, for which data sharing through consortia may play a crucial role. Replication in independent samples is also essential, for which the selection of a single reference group is a critical factor in facilitating the comparability among studies. Besides, studies should provide information on the size of interactions and the effects of gene and dietary exposures separately and in joint effect. Even though it was out of the focus of the current study, recent studies have shown that environmental factors including dietary compounds may modulate gene expression, influence DNA methylation processes, and regulate histone and microRNA assembling, which on the other hand may affect risk of diabetes and cardiovascular disease [90]. Therefore, multi-omics approaches investigating how genetics and epigenetics (and other omics pathways) interact with diet in affecting risk of cardiometabolic diseases should be considered in the future. Finally, the use of prospective data that allows the evaluation of gene-diet interactions effects on incident outcomes should be prioritised.

## Conclusion

Current evidence for gene-diet interaction in CVD is limited, as most interactions have been evaluated in single studies, without multiple correction testing, and mainly in European ethnicities; furthermore, studies have limited information to assess the robustness of sample size. Therefore, data-sharing platforms that combine large studies are needed to address current methodological problems and facilitate replication. In addition, priority should be given to the inclusion of diverse ethnicities and sample size-focused reporting to provide more conclusive evidence of gene-diet interaction with CVD that allows the development of nutritional personalized interventions.

## Supplementary Information

**Additional file 1.** Search strategy.

**Additional file 2.** Supplemental material.

## Data Availability

All data generated or analysed during this study are included in this published article and its supplementary information files.

## Abbreviations

AA: Arachidonic acid
ABCA1: ATP binding cassette subfamily A member 1
ACE: Angiotensin-converting enzyme
ADH1C/ ADH3: Alcohol dehydrogenase 1C
ALA: Alpha-linolenic acid
ALDH2: Aldehyde dehydrogenase 2
APOE: Apolipoprotein E
BCO2: Β-Carotene 9’,10’-oxygenase
CCHS: Copenhagen city heart study
CETP: Cholesteryl ester transfer protein
CGPS: Copenhagen general population study
CHD: Coronary heart disease
CI: Confidence interval
CLOCK: Circadian locomotor output cycles kaput
CRP: C-reactive protein
CVD: Cardiovascular diseases
CYP1A2: Cytochrome P450 family 1 subfamily A member 2
DGAT2: Diacylglycerol O-acyltransferase 2
DHA: Docosahexaenoic acid
ECTIM: Etude cas-témoin de l’infarctus du myocarde
EPA: Eicosapentaenoic acid
EPIC: European prospective investigation into cancer and nutrition
FADS1: Fatty acid desaturase 1
FFQ: Food frequency questionaire
Fgβ: Fibrinogen beta chain
FISSIC: Fangshan/family-based ischemic stroke study in China
Fto/FTO: Fat mass and obesity
GESUS: Danish general suburban population study
GRS: Genetic risk score
GSTs: Glutathione-S-transferase
GSTT1: Glutathione S-transferase theta 1
HDI: Healthy diet indicator
HDL: High-density lipoprotein
HPFS: Health professionals follow-up study
HR: Hazard ratio
IL-6: Interleukin 6
IL-8: Interleukin 8
INTERGENE: Interplay between genetic susceptibility and environmental factors on the risk of chronic diseases in West Sweden
KIHD: Kuopio ischemic heart disease risk factor study
LA: Linoleic acid
LCT: Lactase
LDL: Light density lipoprotein
LPL: Lipoprotein lipase
MDC: Malmö diet and cancer cohort
MDS: Mediterranean diet score
MI: Myocardial infarction
MMAB: Methylmalonic aciduria (cobalamin deficiency) cblB type
MOGAT2: Monoacylglycerol O-acyltransferase 2
MONICA: Multinational monitoring of trends and determinants in cardiovascular disease
MTHFD1: Methylenetetrahydrofolate dehydrogenase, cyclohydrolase and formyltetrahydrofolate synthetase 1
MTHFR: Methylenetetrahydrofolate reductase
MVK: Mevalonate kinase
NIAAA: National Institute on alcohol abuse and alcoholism
NHS: Nurses’ health study
OR: Odds ratio
PDE4D: Phosphodiesterase 4D
PON1: Paraoxonase 1
PUFA: Polyunsaturated fatty acids
RERI: Relative excess risk due to interaction
RFS: Recommended food score
RR: Risk ratio
SFA: Saturated fatty acids
SHEEP: Stockholm heart epidemiology program
SNP: Single nucleotide polymorphism/genetic variant
SSB: Sugar-sweetened beverage
SLC22A3: Solute carrier family 22 member 3
SYTL3: Synaptotagmin like 3
TaqIB: Polymorphism in the cholesteryl ester transfer protein (CETP) gene
TFPI-2: Tissue factor pathway inhibitor-2
TNF: Tumor necrosis factor
TRIB1: Tribbles pseudokinase 1
TWB: Taiwan biobank
VLDL: Very low-density lipoprotein
WENBIT: Western Norway B-vitamin intervention randomised trial
WHS: Women’s health study
β-actin (ACTB): ACTB actin beta
5-LO: 5-Lipoxygenase
