# Supplementary material for: Gene-diet interactions and cardiovascular diseases: a systematic review of observational and clinical trials
Source: BMC Cardiovasc Disord. 2022 Aug 20;22:377. doi: 10.1186/s12872-022-02808-1 (PMC9392936; doi:10.1186/s12872-022-02808-1)
Supplement: Supplementary file 1 — Additional file 1. Search strategy. [file 12872_2022_2808_MOESM1_ESM.docx]

**Gene-diet interactions and cardiovascular diseases: A systematic review of observational and clinical trials**

Zayne M. Roa-Díaz^1,2^, Julian Teuscher^1^, Magda Gamba^1,2^, Marvin Bundo^1,2^, Grisotto Giorgia^1,2^, Faina Wehrli^1^, Edna Gamboa^3^, Lyda Z. Rojas^4^, Sergio Gómez-Ochoa^1^, Sanne Verhoog^5^, Manuel de Jesus Frias Vargas^6^, Beatrice Minder^7^, Oscar H. Franco^1^, Abbas Dehghan^8,9^, Raha Pazoki^10,11,12^, Pedro Marques Vidal^13^, Taulant Muka^1^.

^1^ Institute of Social and Preventive Medicine (ISPM), University of Bern, Bern, Switzerland

^2^ Graduate School for Health Sciences, University of Bern, Bern, Switzerland

^3^ School of Nutrition and Dietetics, Health Faculty, Universidad Industrial de Santander, Bucaramanga, Colombia

^4^ Nursing Research and Knowledge Development Group GIDCEN, Fundación Cardiovascular de Colombia, Floridablanca, Santander, Colombia.

^5^ Erasmus MC, University Medical Center Rotterdam, Department of Public Health

^6^ Centro de Salud Comillas, Madrid, España

^7^ Public Health & Primary Care Library, University Library of Bern, University of Bern, Bern, Switzerland

^8^ Department of Epidemiology, Erasmus MC University Medical Center, Rotterdam, The Netherlands

^9^ Department of Biostatistics and Epidemiology, MRC Centre for Environment and Health, School of Public Health, Imperial College, London, UK

^10^ Department of Life Sciences, College of Health and Life Sciences, Brunel University London, Uxbridge, UK

^11^ MRC Centre for Environment and Health, Department of Epidemiology and Biostatistics, School of Public Health, Imperial College London, London, UK

^12^ CIRTM Centre for Inflammation Research and Translational Medicine, College of Health and Life Sciences, Brunel University London, Uxbridge, UK

^13^ Department of Medicine, Internal Medicine, Lausanne University Hospital (CHUV), University of Lausanne, Lausanne, Switzerland

**Appendix SI.** Search strategy

Combination of concepts: 1) AND 2) AND 3) AND 4) + adding filters 5)

**Medline ALL Ovid**

| **1) Diet, Food, Nutrition** |
| --- |
| (exp "Diet, Food, and Nutrition"/ OR exp Diet/ OR exp Dietary Proteins/ OR Dietary Carbohydrates/ OR exp Dietary Supplements/ OR exp Plant Proteins, Dietary/ OR exp Diet Therapy/ OR exp Dietary Fiber/ OR exp Energy Intake/ OR exp Food/ OR exp Dietary Fats/ OR exp Fatty Acids/ OR diet therapy.fs. OR (diet OR dieting* OR diets OR dietary OR gene-diet* OR (gene adj2 diet) OR nutri* OR macro-nutrient* OR macronutrient* OR micro-nutrient* OR micronutrient* OR nutraceutical* OR food OR dairy OR milk OR ((sugar* OR sucrose* OR fructose* OR soda OR sweet* OR flavor* OR flavour*) adj3 (drink* OR beverage* OR juice* OR sodas)) OR ((sugar* OR sucrose* OR soda OR carbohydrate* OR fat OR caloric OR energy OR vitamin* OR minerals) adj2 (intake OR consumption)) OR fatty acid* OR "saturated fat*" OR "monounsaturated fat*" OR "polyunsaturated fat*" OR PUFA OR MUFA OR "trans fat*" OR omega-3 OR omega-6 OR "linolenic acid*" OR "linoleic acid*" OR alcohol* OR coffee OR tea OR milk OR beer OR wine OR juic* OR eggs OR fruit* OR meat OR nuts OR seeds OR vegetable*).ab,ti,kf) |
| **2) Gene-diet interaction** |
| (Nutrigenomics/ OR Genotype/ OR exp Alleles/ OR Genetic Variation/ OR exp Polymorphism, Genetic/ OR exp Genetic Predisposition to Disease/ OR Epigenomics/ OR (gene-diet* OR (gene adj2 diet*) OR polymorphi* OR "nutritional genomic*" OR nutrigen* OR "nutritional genetic*" OR allele* OR allelic* OR ((genetic) adj3 ("risk score*" OR susceptibilit* OR predisposition* OR association*)) OR epigen*).ab,ti,kf) |
| **3) Outcome: Cardiovascular diseases** |
| (Cardiovascular Diseases/ OR exp Heart Failure/ OR Heart Diseases/ OR Coronary Disease/ OR Coronary Artery Disease/ OR Myocardial Ischemia/ OR exp Atherosclerosis/ OR Carotid Artery Diseases/ OR Cerebrovascular Disorders/ OR exp Stroke/ OR exp Brain Ischemia OR (((cardiovasc* or coronar* or cardio* or cardia* or heart or myocard* or thrombo* or cerebrovasc* or cerebr* or brain) adj3 (disease* or disorder* or event* or failure* or accident* or attack* or insult* or infarct* or ischem* or ischaem* or syndrome* or insufficien* or health* or death*)) or cvd or cvds or cva or stroke* or ((brain or cerebral) adj2 (ischem* or ischaem*)) or cardiopath*).ab,ti,kf.) |
| **4) Strategy to identify study design** |
| (exp Cohort Studies/ OR exp Clinical Trial/ OR exp Case-Control Studies/ OR Cross-Sectional Studies/ OR randomized controlled trial.pt. OR ((cross ADJ section*) OR (case ADJ control*) OR cohort* OR ((prospectiv* OR population* OR observation* OR longitudinal OR retrospect* OR intervent* OR random* OR clinical) ADJ3 (stud* OR trial*)) OR ((Hazard OR odds OR risk*) ADJ (Ratio*)) OR rct* OR randomly OR "follow up").ab,ti,kf.) |
| **5) Limits excluding: studies with child only, animal studies, conference abstracts, letters, notes, editorials, reviews** |
| NOT ((exp child/ OR exp infant/ OR adolescent/) NOT exp adult/)  NOT (exp animals/ NOT humans/) NOT (congresses OR editorial OR guideline OR letter OR news OR published erratum OR review).pt. |

**PubMed**

| **1) Diet, Food, Nutrition** |
| --- |
| ("Diet, Food, and Nutrition"[mh] OR Diet[mh] OR Dietary Proteins[mh] OR Dietary Carbohydrates[mh] OR Dietary Supplements[mh] OR Plant Proteins, Dietary[mh] OR Diet Therapy[mh] OR Dietary Fiber[mh] OR Energy Intake[mh] OR Food[mh] OR Dietary Fats[mh] OR "Fatty Acids"[mh] OR "diet therapy"[Subheading] OR diet[tiab] OR dieting*[tiab] OR diets[tiab] OR dietary[tiab] OR gene-diet*[tiab] OR diet-gene*[tiab] OR nutri*[tiab] OR macro-nutrient*[tiab] OR macronutrient*[tiab] OR micro-nutrient*[tiab] OR micronutrient*[tiab] OR nutraceutical*[tiab] OR food[tiab] OR dairy[tiab] OR milk[tiab] OR ((sugar*[tiab] OR sucrose*[tiab] OR fructose*[tiab] OR soda[tiab] OR sweet*[tiab] OR flavor*[tiab] OR flavour*[tiab]) AND (drink*[tiab] OR beverage*[tiab] OR juice*[tiab] OR sodas[tiab])) OR ((sugar[tiab] OR sucrose*[tiab] OR fructose*[tiab] OR soda[tiab] OR carbohydrate[tiab] OR fat[tiab] OR caloric[tiab] OR energy[tiab] OR vitamin*[tiab] OR minerals[tiab]) AND (intake[tiab] OR consumption[tiab])) OR fatty acid*[tiab] OR "saturated fat*"[tiab] OR "monounsaturated fat*"[tiab] OR "polyunsaturated fat*"[tiab] OR PUFA[tiab] OR MUFA[tiab] OR "trans fat*"[tiab] OR omega-3[tiab] OR omega-6[tiab] OR "linolenic acid*"[tiab] OR "linoleic acid*"[tiab] OR alcohol*[tiab] OR coffee[tiab] OR tea[tiab] OR milk[tiab] OR beer[tiab] OR wine[tiab] OR juic*[tiab] OR eggs[tiab] OR fruit*[tiab] OR meat[tiab] OR nuts[tiab] OR seeds[tiab] OR vegetable*[tiab]) |
| **2) Gene-diet interaction** |
| (Nutrigenomics[mh] OR Genotype[mh] OR Alleles[mh] OR Genetic Variation[mh:noexp] OR "Polymorphism, Genetic"[Mesh] OR Genetic Predisposition to Disease[mh] OR "Epigenomics"[Mesh] OR gene-diet*[tiab] OR diet-gene*[tiab] OR polymorphi*[tiab] OR "nutritional genomic*"[tiab] OR nutrigen*[tiab] OR "nutritional genetic*"[tiab] OR allele*[tiab] OR allelic*[tiab] OR genetic risk score*[tiab] OR genetic susceptibilit*[tiab] OR genetic association*[tiab] OR genetic predisposition*[tiab] OR epigen*[tiab]) |
| **3) Outcome: Cardiovascular diseases** |
| (Cardiovascular Diseases[mh] OR Heart Failure[mh] OR Heart Diseases[mh] OR Coronary Disease[mh] OR Coronary Artery Disease[mh] OR Myocardial Ischemia[mh] OR Atherosclerosis[mh] OR Carotid Artery Diseases[mh] OR Cerebrovascular Disorders[mh] OR Stroke[mh] OR Brain Ischemia OR ((cardiovasc*[tiab] or coronar*[tiab] or cardio*[tiab] or cardia*[tiab] or heart[tiab] or myocard*[tiab] or thrombo*[tiab] or cerebrovasc*[tiab] or cerebr*[tiab] or brain[tiab]) AND (disease*[tiab] or disorder*[tiab] or event*[tiab] or failure*[tiab] or accident*[tiab] or attack*[tiab] or insult*[tiab] or infarct*[tiab] or ischem*[tiab] or ischaem*[tiab] or syndrome*[tiab] or insufficien*[tiab] or death*[tiab])) or cvd[tiab] or cvds[tiab] or cva[tiab] or stroke*[tiab] or ((brain[tiab] or cerebral[tiab]) AND (ischem*[tiab] or ischaem*[tiab])) or cardiopath*[tiab]) |
| **4) Strategy to identify study design** |
| (Cohort Studies[mh] OR Clinical Trial[mh] OR Case-Control Studies[mh] OR Cross-Sectional Studies[mh] OR randomized controlled trial[pt] OR cross-section*[tiab] OR case-control*[tiab] OR cohort*[tiab] OR ((prospectiv*[tiab] OR population*[tiab] OR observation*[tiab] OR longitudinal[tiab] OR retrospect*[tiab] OR random*[tiab] OR intervent*[tiab] OR clinical[tiab]) AND (stud*[tiab] OR trial*[tiab])) OR ((Hazard[tiab] OR odds[tiab] OR risk*[tiab]) AND (Ratio*[tiab])) OR rct[tiab] OR randomly[tiab] OR "follow up"[tiab]) |
| **5) Limits excluding: studies with child only, animal studies, conference abstracts, letters, notes, editorials, reviews** |
| NOT ((child[mh] OR infant[mh] OR adolescent[mh]) NOT adult[mh])  NOT (animals[mh] NOT humans[mh]) NOT (letter[pt] OR news[pt] OR comment[pt] OR published erratum[pt] OR editorial[pt] OR congress[pt] OR guideline[pt] OR review[pt]) |

**Embase.com**

| **1) Diet, Food, Nutrition** |
| --- |
| ('diet'/exp OR 'protein intake'/de OR 'carbohydrate intake'/exp OR 'dietary supplement'/de OR 'diet therapy'/exp OR 'dietary fiber'/exp OR 'fiber intake'/de OR 'dietary intake'/exp OR 'caloric intake'/de OR 'food intake'/exp OR 'food'/exp OR 'fat intake'/exp OR 'fatty acid'/exp OR 'nutrition'/exp OR 'vegetable'/exp OR (diet OR dieting* OR diets OR dietary OR gene-diet* OR (gene NEAR/2 diet) OR nutri* OR macro-nutrient* OR macronutrient* OR micro-nutrient* OR micronutrient* OR nutraceutical* OR food OR dairy OR milk OR ((sugar* OR sucrose* OR fructose* OR soda OR sweet* OR flavor* OR flavour*) NEAR/3 (drink* OR beverage* OR juice* OR sodas)) OR ((sugar* OR sucrose* OR soda OR carbohydrate* OR fat OR caloric OR energy OR vitamin* OR minerals) NEAR/2 (intake OR consumption)) OR fatty acid* OR 'saturated fat*' OR 'monounsaturated fat*' OR 'polyunsaturated fat*' OR PUFA OR MUFA OR 'trans fat*' OR omega-3 OR omega-6 OR 'linolenic acid*' OR 'linoleic acid' OR alcohol* OR coffee OR tea OR milk OR beer OR wine OR juic* OR eggs OR fruit* OR meat OR nuts OR seeds OR vegetable*):ab,ti,kw) |
| **2) Gene-diet interaction** |
| ('gene diet interaction'/de OR 'nutrigenomics'/de OR 'nutriomics'/exp OR 'genotype environment interaction'/de OR 'allele'/exp OR 'genetic variation'/de OR 'genetic variability'/de OR 'genetic association'/exp OR 'genetic polymorphism'/exp OR 'DNA polymorphism'/de OR 'genetic predisposition'/exp OR 'genetic risk score'/de OR 'epigenetics'/exp OR (gene-diet* OR (gene NEAR/2 diet*) OR polymorphi* OR 'nutritional genomic*' OR nutrigen* OR 'nutritional genetic*' OR allele* OR allelic* OR ((genetic) NEAR/3 ('risk score*' OR susceptibilit* OR predisposition* OR association*)) OR epigen*):ab,ti,kw) |
| **3) Outcome: Cardiovascular diseases** |
| ('cardiovascular disease'/de OR 'cardiovascular event'/de OR 'cardiovascular mortality'/de OR 'heart failure'/exp OR 'heart disease'/de OR 'coronary artery disease'/de OR 'ischemic heart disease'/exp OR 'atherosclerosis'/de OR 'coronary artery atherosclerosis'/de OR 'cerebrovascular accident'/exp OR 'brain ischemia'/exp OR 'atherosclerotic cardiovascular disease'/de OR (((cardiovasc* OR coronar* OR cardio* OR cardia* OR heart OR myocard* OR thrombo* OR cerebrovasc* OR cerebr* OR brain) NEAR/3 (disease* OR disorder* OR event* OR failure* OR accident* OR attack* OR insult* OR infarct* OR atheroscler* OR arterioscler* OR ischem* OR ischaem* OR syndrome* OR insufficien* OR health* OR death*)) OR cvd OR cvds OR cva OR stroke* OR ((brain OR cerebral) NEAR/3 (ischem* OR ischaem*)) OR cardiopath*):ab,ti,kw) |
| **4) Strategy to identify study design** |
| ('cohort analysis'/de OR 'prospective study'/de OR 'follow up'/de OR 'longitudinal study'/de OR 'retrospective study'/de OR 'case control study'/exp OR 'intervention study'/de OR 'clinical study'/de OR 'clinical trial'/exp OR 'cross-sectional study'/de OR 'major clinical study'/de OR ((cross NEXT/1 section*) OR (case NEAR/3 control*) OR cohort* OR ((prospectiv* OR population* OR observation* OR longitudinal OR retrospect* OR intervent* OR clinical OR random*) NEAR/3 (stud* OR trial*)) OR ((Hazard OR odds OR risk*) NEXT/1 (Ratio*)) OR rct* OR randomly OR 'follow up'):ab,ti,kw) |
| **5) Limits excluding: studies with child only, animal studies, conference abstracts, letters, notes, editorials, reviews** |
| NOT (([infant]/lim OR [child]/lim OR [adolescent]/lim) NOT [adult]/lim)  NOT ([animals]/lim NOT [humans]/lim) NOT ([Conference Abstract]/lim OR [Letter]/lim OR [Note]/lim OR [Editorial]/lim OR [Review]/lim) |

**Cochrane CENTRAL**Cochrane Central Register of Controlled Trials

| **1) Diet, Food, Nutrition** |
| --- |
| ((diet OR dieting* OR diets OR dietary OR (gene NEXT diet*) OR (gene NEAR/2 diet*) OR nutri* OR (macro NEXT nutrient*) OR macronutrient* OR (micro NEXT nutrient*) OR micronutrient* OR nutraceutical* OR food OR dairy OR milk OR ((sugar* OR sucrose* OR fructose* OR soda OR sweet* OR flavor* OR flavour*) NEAR/3 (drink* OR beverage* OR juice* OR sodas)) OR ((sugar* OR sucrose* OR fructose* OR soda OR carbohydrate* OR fat OR caloric OR energy OR vitamin* OR minerals) NEAR/2 (intake OR consumption)) OR (fatty NEXT acid*) OR (saturated NEXT fat*) OR (monounsaturated NEXT fat*) OR (polyunsaturated NEXT fat*) OR PUFA OR MUFA OR (trans NEXT fat*) OR omega-3 OR omega-6 OR (linolenic NEXT acid*) OR (linoleic NEXT acid) OR alcohol* OR coffee OR tea OR milk OR beer OR wine OR juic* OR eggs OR fruit* OR meat OR nuts OR seeds OR vegetable*):ab,ti,kw) |
| **2) Gene-diet interaction** |
| (((gene NEXT diet*) OR (gene NEAR/2 diet*) OR polymorphi* OR (nutritional NEXT genomic*) OR nutrigen* OR allele* OR allelic* OR (genetic NEXT risk NEXT score*) OR ((genetic) NEAR/3 (susceptibilit* OR predisposition* OR association*)) OR epigen*):ab,ti,kw) |
| **3) Outcome: Cardiovascular diseases** |
| ((((cardiovasc* or coronar* or cardio* or cardia* or heart or myocard* or thrombo* or cerebrovasc* or cerebr* or brain) NEAR/3 (disease* or disorder* or event* or failure* or accident* or attack* or insult* or infarct* or atheroscler* or arterioscler* or ischem* or ischaem* or syndrome* or insufficien* or health* or death*)) or cvd or cvds or cva or stroke* or ((brain or cerebral) NEAR/3 (ischem* or ischaem*)) or cardiopath*):ab,ti,kw) |
