# Supplementary material for: Gene-diet interactions and cardiovascular diseases: a systematic review of observational and clinical trials
Source: BMC Cardiovasc Disord. 2022 Aug 20;22:377. doi: 10.1186/s12872-022-02808-1 (PMC9392936; doi:10.1186/s12872-022-02808-1)
Supplement: Supplementary file 2 — Additional file 2. Supplemental material. [file 12872_2022_2808_MOESM2_ESM.docx]

**Gene-diet interactions and cardiovascular diseases: A systematic review of observational and clinical trials**

Zayne M. Roa-Díaz^1,2^, Julian Teuscher^1^, Magda Gamba^1,2^, Marvin Bundo^1,2^, Grisotto Giorgia^1,2^, Faina Wehrli^1^, Edna Gamboa^3^, Lyda Z. Rojas^4^, Sergio Gómez-Ochoa^1^, Sanne Verhoog^5^, Manuel de Jesus Frias Vargas^6^, Beatrice Minder^7^, Oscar H. Franco^1^, Abbas Dehghan^8,9^, Raha Pazoki^10,11,12^, Pedro Marques Vidal^13^, Taulant Muka^1^.

^1^ Institute of Social and Preventive Medicine (ISPM), University of Bern, Bern, Switzerland

^2^ Graduate School for Health Sciences, University of Bern, Bern, Switzerland

^3^ School of Nutrition and Dietetics, Health Faculty, Universidad Industrial de Santander, Bucaramanga, Colombia

^4^ Nursing Research and Knowledge Development Group GIDCEN, Fundación Cardiovascular de Colombia, Floridablanca, Santander, Colombia.

^5^ Erasmus MC, University Medical Center Rotterdam, Department of Public Health

^6^ Centro de Salud Comillas, Madrid, España

^7^ Public Health & Primary Care Library, University Library of Bern, University of Bern, Bern, Switzerland

^8^ Department of Epidemiology, Erasmus MC University Medical Center, Rotterdam, The Netherlands

^9^ Department of Biostatistics and Epidemiology, MRC Centre for Environment and Health, School of Public Health, Imperial College, London, UK

^10^ Department of Life Sciences, College of Health and Life Sciences, Brunel University London, Uxbridge, UK

^11^ MRC Centre for Environment and Health, Department of Epidemiology and Biostatistics, School of Public Health, Imperial College London, London, UK

^12^ CIRTM Centre for Inflammation Research and Translational Medicine, College of Health and Life Sciences, Brunel University London, Uxbridge, UK

^13^ Department of Medicine, Internal Medicine, Lausanne University Hospital (CHUV), University of Lausanne, Lausanne, Switzerland

**Table S1.** Categories of food (1)

| Food categories | |
| --- | --- |
| Macronutrients | - Carbohydrates - Lipids, sterols and their metabolites - Proteins and aminoacids |
| Micronutrietns | - Vitamins   - water-soluble vitamins: thiamine, riboflavin, niacin, vitamine B6, biotin, Pantotenic acid, Folic acid, Cobalamine (b12), Vitamin C, Coline   - fat-soluble vitamins: A, D, E, K - Minerals:   - Major minerals: Sodium, Potassium, Chloride, Calcium, Phosphorus, Magnesium, Sulfur   - trace elements: Iron, Copper, Zinc, Iodine, Selenium, Manganese, chromium, fluoride, molybdenum |
| Other dietary components | - Foods that have no caloric intake or are not indispensable in the diet (fiber, alcohol, beverages, sugar-sweetened beverages-juice) |
| Food groups or categories | - Milk and dairy products - Meat, poultry and fish - Egg - Fruits - Vegetables - Cereals and pseudocereals:   - Cereals: maize, rice, wheat, barley, Sorghum, Millet, oats, triticale, rye.   - Pseudocereals: amaranth, quinoa, hanza, chia, breadnut, buckwheat,   - Cereal derivatives: all kinds of products produced from cereals (bread, cookies, cakes) - Pulses and edible nuts:   - Pulses: alfalfa, beans, soybeans, peas, chickpeas, lentils, lupins, peanuts   - Nuts: almond, brazil nut, cashew, chestnuts, hazelnuts, macadamia, pistachio, walnuts - Tubers, plantains and roots (high in carbohydrates)   - Tubers: potatoes, yam,   - Plantains: cooking banana (sweet and green)   - Roots: Arracacha, parsnip, yuca or cassava - Cooking and seasoning oils - Sugars: Cain sugar (white, brown), honey, plant syrups - Water and other beverages |
| Empirically-derived dietary patterns, dietary patterns and dietary score | Reported by studies. |

Table S2. complementary information of the included articles

| Author | Outcome definition | Outcome measurement | Amount intake/ description of the interactor diet | (Gene/chromosome region/GRS) | Allele frequency | Nearest gene function | women main findings | Funding |
| --- | --- | --- | --- | --- | --- | --- | --- | --- |
| Myocardial infarction | | | | | | | | |
| Allayee H et al, 2008 | WHO criteria for MI, which requires typical symptoms plus either elevation in cardiac enzyme concentrations or diagnostic changes in the electrocardiogram. | Medical confirmation | Low/ high intake of arachidonic acidFood-frequency questionnaire developed and validated to assess fatty acid intake | 5-LO | common promoter allele consists of 5 repeats and has a frequency of ≈0.80, whereas alleles of4, 3, and longer than 5 repeats (ie, 6, 7, 8) ≈0.15 | 5-Lipoxygenase (5-LO) associated with atherosclerosis in different animals models and in humans. | No | National Institutes of Health, American Heart Association and National Center for Research Resources |
| Chen Q et al, 2017 | MI diagnosed by either ST elevation, according blood markers or typical symptoms. | Medical confirmation | Alcohol consumption more or less than 250g. per day. | PCSK9 | The minor C allele was 12.17% in the AMI group, in the control group was 6.50% for the minor C alleles, respectively | Inhibiton of PCSK9 is used as an effecitve treatment to hyperlipidemia and polymorphisms in this gene are associated with individual differences in cardiovascular risk profile. | No | Supported by the National Natural Science Foundation of China and others. |
| Cornelis M et al, 2007 | WHO criteria for MI, which require typical symptoms plus either an elevation in cardiac enzyme concentrations or a diagnostic change on electrocardiogram. | Medica confirmation | Servings per day. Intakes of nutrients were calculated by using the US-Department of Agriculture food composition data file | GSTT1, GSTP1, GSTM1 | NR | Gluthation-S-Transferase (GSTs) are a super-famiIly of xenobiotic-metabolising enzymes that generally “detoxify” reactive metabolites to more water-soluble and readily excretable forms. | No | NR |
| Cornelis M et al, 2006 | WHO criteria for MI, which require typical symptoms plus either elevation in cardiac enzyme levels or diagnostic change on electrocardiogram. | Medical confirmation | Cups (250ml) per daySemi-quantitative food frequency questionnaire (FFQ) | CYP1A2 | *1F allele 54% | CYP1A2 encodes the CYP 450 1A2 enzyme which metabolises roughly 95% of caffeine in the liver, polymorphisms of this gene result in different activities in caffein metabolism. | No | This research was supported by grants from the Canadian Institutes of Health Research and the National Institutes of Health |
| Ding Y et al, 2016 | Fatal or non-fatal MI | Medical confirmation | B6 alone, B12 alone, B6 and B12 together NR | MTHFD1 | MAF of the MTHFD1 polymorphism was 42.7% | Methylenetetrahydrofolate dehydrogenase catalyses three different reactions in the folate cycle. Polymorphisms in this gene have been linked to impaired lipid metabolism and oxidative stress. | No | University of Bergen, the Department of Heart Disease, Haukeland University Hospital, Norway, the Western Norway Health Authority, and the Foundation to Promote Research into Functional VitMIn B12 Deficiency. |
| Fumeron F et al, 1995 | MI Survivors | Medical confirmation | Grams per day | CETP | CETP/TaqIB 2 40% | CETP is a plasma-protein which is involved in the transfer of cholesterin ester from HDL to other lipoproteins. | No | NR |
| Hartiala J et al, 2012 | WHO criteria for MI, which requires typical symptoms plus either elevation in cardiac enzyme concentration or diagnostic changes in the electrocardiogram. | Medical confirmation | High/ low omega 6 fatty acids, high/ low omega 3 fatty acids FFQ | PLA2G4A | frequency of the G allele was ;14% | PLA2G4A catalyzes the hydrolysis of membrane phospholipids to release arachidonic acid which gets metabolized into eicosanoids and other pro-inflammatory mediators. | No | NIH USA |
| Hines L et al, 2001 | WHO criteria | Medical records | Drinkes per week, drinks per day | ADH1C | ADH3 alleles among the control subjects in this study population were 60 percent for the g 1 allele and 40 percent for the g 2  allele | ADH catalyses the reaction af ethanol to ethanal/ acetaldehyde, different polymorphsims in the ADH1 gene are linked to different rates of metabolising of alcohol. | No | NR |
| Li J et al, 2020 | MI according to the guidelines of the European Resuscitation Council | Medical records | Alcohol intake per day, categorised as more or less than 250g. /dayFood Questionnaire on the preceeding 12 months prior to event. | CONNEXIN 37 | NR | CONNEXIN37 plays a part in regulation of cellular aging and the regeneration of damaged tissues. | Yes | This study was funded by the National Natural Science Foundation of China |
| Tolstrup J et al, 2009 | WHO criteria, ICD-8 410 and ICD-10 I21 and I22 | Registries | Alcohol intake as drinks per week | ADH1C/ ADH1B | NR | ADH catalyses the reaction af ethanol to ethanal/ acetaldehyde, different polymorphsims in the ADH1 gene are linked to different rates of metabolising of alcohol. | No | Danish Graduate Schoolof Public Health, Danish Heart Foundation, Chief Physician Johan Boserup and Lise Boserup Foundation, Health Insurance Foundation, Ministry of the Interior and Health, Danish Cancer Society, Danish National Board of Health |
| Trichopolou A. et al, 2008 | Coronary infarct (International Classification ofDiseases, Ninth Revision, code 410, and International Statistical Classification of Diseases and Health-Related Problems, Tenth Revision, code 121) | Medical confirmation | High rsik/ low riskMediterranean diet-score | APOA5, APOC3, APOE, IL1β, IL6, LPL, MTHFR, NOS3, and TNF GRS-MI | NR | - | No | Europe Against Cancer Program of the European Commission; the Greek Ministries of Health and Education; a grant to the Hellenic Health Foundation by the Costopoulos Foundation; and grants HL54776 and 58-1950-9-001 from the US Department of Agriculture Research Service, National Heart, Lung, and Blood Institute, National Institutes of Health. |
| Wang F et al, 2019 | According to guidelines of the European Resuscitation Council | Clinical examination | Alcohol intake: less than 250g. / day, more than 250 g. / daynInterview | CXCL12 | The C allele frequency was 77.33% in the AMI group, in the control group was 75.00%. | CXC-Motiv-Chemokin 12 is a chemokine involved in various inflammatory processes, there are reports suggesting association of CXCL12 with increased susceptibility to myocardial ischemia. | No | NR |
| Zheng Y et al, 2016 | WHO criteria for MI, which require typical symptoms plus either elevations in cardiac enzyme concentrations or diagnostic changes in an electrocardiograph | Medical confirmation | Less than 1 serving/day, 1-2 servings/day, more than 2 servings/dayFood frequency questionnaire | CDKN2B-AS1 | All the 3 chromosome 9p21 SNPs were commonly distributed  in the current study participants, with minor allele frequencies that ranged from 0.41 to 0.50 | This gene is located within the CDKN2B-CDKN2A gene cluster at chromosome 9p21. This region is a significant genetic susceptibility locus for cardiovascular disease, and has also been linked to a number of other pathologies. | No | NIH USA |
| Coronary Heart Disease | | | | | | | | |
| Bos M M et al, 2021 | CAD which we defined as: angina pectoris, acute myocardial infarction, and acute and chronic ischemic heart disease. | NHS record systems. | Groups of oily fish intake only once, twice, or three or more times per week | APOE | NR | APOE is an important apolipoprotein and a major component of chylomicrones. It binds to a specific liver and peripheral cell receptor, and is essential for the normal catabolism of triglyceride-rich lipoprotein constituents. | Yes | European Commission and Dutch Heart Foundation |
| Chen H et al, 2018 | CAD cases were defined as followings: at least one of the major segments of coronary arteries (right coronary artery, left circumflex, or left anterior descending arteries) with more than or equal to 50% organic stenosis based on coronary angiography | Coronary Angiography | Never/ Current drinker | Il6 | The frequency for the C allele of rs1800795 was significantly  higher in CAD patients than controls (38.46% vs 26.71%) | This gene encodes a cytokine that functions in inflammation and the maturation of B cells. The functioning of this gene is implicated ininflammation-associated disease states, including suspectibility to diabetes mellitus. | No |  |
| Chi Y et al, 2018 | Coronary angiography (> 50% diameter stenosis in at least one of the major coronary arteries) | Medical confirmation | Drinkers = 1 or more drink per month, non-drinkers = less than 1 drink per month | PLA2G7 | rs1805017 Allele, H (30.4%)  rs16874954 Allele, F (28.1%)  rs1805018 Allele, T (31.2%)  rs1051931 Allele, V (25.8%) | Lipoprotein-associated phospholipase A2 is assumed to be associated with cardiovascular risk as an individual inflammatory property. | No |  |
| Corella D et al, 2010 | Incident definite fatal or non-fatal myocardial infarction or angina requiring a revascularization procedure. | Medical records and AHA guidelines | Drinker/ non-drinker Validated computerized diet | CETP | NR | CETP is a plasma-protein which is involved in the transfer of cholesterin ester from HDL to other lipoproteins. | No | Spain Ministry of Health |
| Ebrahim S et al, 2008 | WHO diagnostic criteria | Medical confirmation and linkage to register | Alcohol intake as units/ week | ADH1C | NR | ADH catalyses the reaction af ethanol to ethanal/ acetaldehyde, different polymorphsims in the ADH1 gene are linked to different rates of metabolising of alcohol. | Yes | Department of Health and British Heart Foundation, UK Department of Health Career ScientistAward, UK Medical Research Councilstudentship and Medical Research Council of the United Kingdom |
| Gustavsson J et al, 2016 | Incident first-time acute myocardial infarction (MI) who survived at least 28 days | Medical confirmation | Mediterranean diet / Food frequency questionnaire | FTO | NR | Fat mas and obesity-associated gene (FTO). In both adults and children, FTO risk variants have been associated with impaired satiety responsiveness. FTO knockout mice have a reduction in both fat mass and lean mass, increased metabolic rate and food intake, and decreased propensity to gain weight on a high-fat diet. | No | NR |
| Heidrich J et al, 2007 | Nonfatal and fatal CHD cases | Registry | Mediterranean diet / Food frequency questionnaire | ADH1C | NR | ADH catalyses the reaction af ethanol to ethanal/ acetaldehyde, different polymorphsims in the ADH1 gene are linked to different rates of metabolising of alcohol. | Yes | Studies were initiated and financed by theGSF – National Research Centre for Environment and Health which is funded by the German National Genome Research Network |
| Huang L et al, 2018 | - | Medical records | Never (<once a week) / regular (>1 to 4 times per week) | ALDH2 | NR | ADH catalyses the reaction af ethanol to ethanal/ acetaldehyde, different polymorphsims in the ADH1 gene are linked to different rates of metabolising of alcohol. | No | Zhejiang Leading Team of Science and Technology Innovation (no. 2011R50021) and Zhejiang Provincial Natural Science Foundation of China (no. LQ16H260002). |
| Jensen M et al, 2008 | Incident CHD, defined as non-fatal MI and fatal CHD. | Autoreport and medical records | Mediterranean diet / Food frequency questionnaire | CETP | NR | CETP is a plasma-protein which is involved in the transfer of cholesterin ester from HDL to other lipoproteins. | Yes | Grants from National Institutes of Health, Bethes, Danish Research Foundation, Wedell-Wedellsborg foundation and the Danish Cardiovascular Research Academy |
| Liu F et al, 2015 | WHO 1999/2000 guideline CAD [International Classification of Diseases -10 codesI20-I25] | Medical records | Mediterranean diet / Food frequency questionnaire | FADS1 | NR | Desaturase enzymes of the Fatty acid desaturases (FADS) cluster regulate unsaturation of fatty acids through the introduction of double bonds between defined carbons of the fatty acyl chain. | No | NR |
| Liu Y et al, 2020 | CHD was defined according to ICD-9 codes (410–414) | Self-report, medical confirmation | Regular coffe drinkers vs. non-coffee drinkers | TRIB1 | NR | TRIB1 gene is involved in cholesterol metabolism and is associated with the development of atheroscerosis. | No | Ministry of Science and Technology of Taiwan (MOST) |
| Mehlig K. et al.2014 | Myocardial infarction according to (ICD 10: I21.0, I21.9, I20.0), typical history, ECG, and enzyme changes or un-stable angina | Medical confirmation | Mediterranean diet / Food frequency questionnaire | CETP | NR | CETP is a plasma-protein which is involved in the transfer of cholesterin ester from HDL to other lipoproteins. | Yes | Swedish Research Council, Swedish Heart-Lung Foundation and Swedish council for working life and social research |
| Tolstrup J et al, 2010 | Unstable angina, fatal and non-fatal myocardial infarction | Registries | Mediterranean diet / Food frequency questionnaire | ADH1B/ ADH1C | Allele frequencies were 0.98 (95% CI: 0.97–0.98) (ADH1B.1 slow), 0.02 (95% CI: 0.02–0.03) (ADH1B.2 fast) and 0.61 (95% CI: 0.59–0.62) (ADH1C.1,fast) and 0.39 (95%CI: 0.38–0.41) (ADH1C.2,slow). | ADH catalyses the reaction af ethanol to ethanal/ acetaldehyde, different polymorphsims in the ADH1 gene are linked to different rates of metabolising of alcohol. | No | Support by axel Muusfeldt Foundation, Danish Cancer Society, Svend Andersen’s Fond and the grant IMAGE from theDanish Ministry of Health, Research Centre forEnvironmental Health’s Fund |
| Virtanen J et al, 2016 | Coronary event (International Classification of Diseases, 9th revision, codes 410–414 and International Classification of Diseases, 10th revision, codes I20–I25) | Registries | Mediterranean diet / Food frequency questionnaire | APOE4 | NR | APOE is an important apolipoprotein and a major component of chylomicrones. It binds to a specific liver and peripheral cell receptor, and is essential for the normal catabolism of triglyceride-rich lipoprotein constituents. | No | NR |
| Yiannakouris N et al, 2014 | CHD events included myocardial infarction, angina and other ischaemic heart disease (cardiac arrest, presence of cardiac and vascular implants and grafts) | Medical confirmation | Mediterranean diet / Food frequency questionnaire. MedDiet score incorporates the salient characteristics of this diet (high intake of plant foods and olive oil, low intake of meat and dairy products and moderate intake of alcohol). This score, with values from 0 to 9 (higher scores indicate greater adherence to the MedDiet) | PCSK9, CELSR2-PSRC1-SORT1, MIA3, WDR12, PHACTR1, CXCL12, LDLR, SLC5A3-MRPS6-KCNE2, CDKN2A/2B | NR | - | No | Hellenic Health Foundation and the Stavros Niarchros Foundation; and by contracts 53-K06-5-10 and 58-1950-9-001 from the US Department of Agriculture Research |
| Younis J et al, 2005 | - | - | Mediterranean diet / Food frequency questionnaire | ADH1C | Rare allele frequency in abstainer 0.373 (95% CI 0.34–0.40) and consumers 0.410 (95% 0.40–0.42) | ADH catalyses the reaction af ethanol to ethanal/ acetaldehyde, different polymorphsims in the ADH1 gene are linked to different rates of metabolising of alcohol. | No |  |
| Zhou H et al, 2019 | WHO criteria for CAD set in 1979 | Clinical examination - Medical confirmation | Mililiters per week | TFPI-2 | rs59805398 Allele, G (23.5% and 28.2%)  rs34489123 Allele, A (20.4% and 31.5%)  rs4264 Allele, G (19.7% and 29.8%)  rs4271 Allele, T (21.0% and 24.8%) | Studies suggest that TFPI2 could present an antiangiogenic factor and could contribute to aberrant angiogenesis. | No |  |
| Mukamal K et al, 2009 | Incident CHD, defined as non-fatal myocardial infarction (MI) or fatal CHD | Self-report and medical records | Alcohol intake as g./ day, more than 2.5g./ day, less than 2.5g./ day1-week dietary record | PON1 | NR | Paraoxonase 1 is an endogenous antiatherogenic protein. It binds to high density lipoprotein (HDL) particles and hydrolyzes thiolactones and xenobiotics. Polymorphisms in this gene may be associated with coronary artery disease and diabetic retinopathy. | Yes | National Institutes of Health |
| Stroke | | | | | | | | |
| Mukamal K et al, 2005 | To be categorized as a stroke, a new neurologic deficit had to persist for 24 hours, or imaging studies had to demonstrate a lesion appropriate to the clinical deficit | Medical confirmation, medical records. | Alcohol intake per week. Yearly standardized questionnaires | APOE | NR | APOE is an important apolipoprotein and a major component of chylomicrones. It binds to a specific liver and peripheral cell receptor, and is essential for the normal catabolism of triglyceride-rich lipoprotein constituents. | No |  |
| Chen Z et al, 2015 | WHO criteria | All patients were diagnosed by CT and MRI within 48 hours after the admission, ultrasound, brain color Doppler, CT angiography and magnetic resonance angiography | NR | CRP | rs3093059, allele C 17%  rs1800947, allele C 5.3% | C-reactive protein (CRP) is an acute-phase-protein involved in defense related functions. CRP recognize foreign pathogens and damaged cells of the host and initiate their elimination by interacting with humoral and cellular effector systems. CRP levels are elevated during acute phase infection or other inflammatory stimuli. | No | Grants from the Aerospace Science and industry group project |
| Gao X et al, 2006 | International Classification of Disease (ninth revision) | Clinical examination - Medical confirmation | NR | FgB | t allele of FgB in the case group (30%) in the control group (19.5%) | Fibrinogen beta chain is part fibrinogen, a blood-borne glycoprotein comprised of three pairs of nonidentical polypeptide chains. Fibrinogen is the most abundant component of blood clots, built after vascular injury. | No |  |
| Juan J et al, 2017 | Neurological deficits of sudden or rapid onset lasting for greater than 24 h or until death due to thrombotic or embolic occlusion of a cerebral artery | Self-report, medical history, physical examinations, laboratory tests and results of CT and/or MRI, medical confirmation | Intake as g./ dayQuestionnaires were a shortened version of full sFFQ from Shanghai Women’s Health Study (SWHS) | PON1 | NR | Paraoxonase 1 is an endogenous antiatherogenic protein. It binds to high density lipoprotein (HDL) particles and hydrolyzes thiolactones and xenobiotics, including paraoxon. Polymorphisms in this gene may be associated with coronary artery disease and diabetic retinopathy. | No | Supported by grants from the Key Project of Natural Science Funds of China,the National Natural Science Foundation of China and Beijing Natural Science Foundation |
| Kamdee K et al, 2021 | NR | Medical using clinical symptoms, computed tomography scans and/or magnetic resonance imaging | Alcohol drinkers were classified as people consuming ≥100 ml alcohol >3 times per week. | IL6 and TNF‑α | MAF IL‑6 cases 2.5% and controls 3.9%. MAF TNF‑α cases 9.3% and controls 4.8%. | IL6 encodes a cytokine that functions in inflammation and the maturation of B cells. The functioning of this gene is implicated ininflammation-associated disease states, including suspectibility to diabetes mellitus. TNF is a gene that encodes a multifunctional proinflammatory cytokine that belongs to the tumor necrosis factor (TNF) superfamily | No | Walailak University Fund, hailand Science Research and Innovation Fund and Development and Promotion of Science and Technology Talents Project |
| Luo S et al, 2013 | Cerebral infarction as defined by WHO | Medical records and MRI or CT. | Drinkers vs. non drinkers, drinking defined as more than 15g. ethanol / day- | Il8 | NR | IL8 plays an important role in inflammatory processes and the pathogenesis of atherosclerosis. | No | The Project of Guangdong Provincial Natural Science Foundation |
| Song J et al, 2019 | Brain infarction due to occlusion of precerebral arteries (international classification of diseases [ICD]-9 433), brain infarction due to cerebral thrombosis (ICD-9 434), and embolic brain infarction (ICD-9 434) | Medical confirmation, and head CT or MRI | 4 eggs per week | ABCA1 | NR | ATP-binding-cassette A1/ ABCA1 is a transporter for cholesterol and plays a vital role in the cellular lipid removal pathway. | No | NR |
| Yang S et al, 2020 | - | Record linkage/ household and community survey | Alcohol intake, categorised in never-drinkers, light to moderate drinkers and heavy drinkers (more than 1 drink a day for women, more than 2 drinks a day for men) | ACTB | Major/minor  rs852426 T/C 3130/894 and 3460/952  rs852423 A/G  2795/1227 3107/1311  rs2966449 T/C  3033/991 3323/1091 | ACTB gene encodes for beta-actin a cytoskeleton protein with diverse regulatory functions in division, growth and organisation of the cytoskeleton. Polymorphism in the gene are associated with vascular hypertrophy, hypertension and cerebral infarction. | No | NR |
| Zhang L et al, 2019 | - | Medical confirmation, and head CT or MRI | Drinkers vs. non drinkers, drinkers are persons who drink alkohol every week over the duration of one year. | CONNEXIN 37 and PDE4D | CONNEXIN37–rs1764391 T allele (19.5% - 28.9%);  CONNEXIN37–rs1764390 (22.9%-25%); G allele  PDE4D gene–rs966221  (SNP83) (20.7%; 29.1);G allele  PDE4D gene–rs918592 (SNP87) T allele (23.3%-27.8%) | CONNEXIN37 plays a part in regulation of cellular aging and the regeneration of damaged tissues. PDE4D encodes for phosphodiesterase 4 an enzyme that degrades the second messenger cAMP and thus plays a vital role in cellular information cycles. | No | NR |
| Zhao T et al, 2019 | IS according to Criteria from "Society of Neurology, Chinese Medical Association" | Clinical examination - Medical confirmation | Inadequate fruit/ vegetable intake= less than 300g. per day, adequate fruit/ vegetable intake= more than 300g. per day | BCO2 | The minor allele frequencies (MAFs) of these five SNPs were greater than 5% | β-carotene 9',10'-oxygenase plays an important role in lipid and cholesterol metabolism. | No | Supported by the Program for Zhejiang Leading Team of Science and Technology Innovation |
| Zhou YG et al, 2021 | IS was diagnosed according to the Trial ofOrg 10,172 in Acute Stroke Treatment (TOAST) criteria | Clinical examiantion- Medical confirmation | Alcohol consumption was categorized into subgroups ofgrams ofalcohol per day: 0 (non-drinker), ≤25 and >25. | DGAT2 and MOGAT2 | The minor allele frequency (MAF) of the SNPs was higher than 5%; | DGAT2 encodes one of two enzymes which catalyzes the final reaction in the synthesis of triglycerides and MOGAT2 encodes a protein that catalyzes the synthesis of diacylglycerol from 2-monoacylglycerol and fatty acyl-CoA | No | National Natural Science Foundation of China and Youthful Science Foundation ofGuangxi Province |
| Zheng X et al, 2020 | - | Medical confirmation, and head CT or MRI | NR | MTHFR | The frequency of the rs4846049-A allele was 28.6% in IS patients and 19.1% in normal controls, in addition, the frequency of the rs3737967-T allele was 27.9% in IS patients and 20.3% in normal controls, which was also indicating a statistically significant difference | Methylenetetrahydrofolate reductase plays an important role in the folate cycle and catalyses the conversion of 5,10-methylenetetrahydrofolate to 5-methyltetrahydrofolate, a co-substrate for homocysteine remethylation to methionine. | No | None |
| Cardiovascular disease | | | | | | | | |
| Djousse L et al, 2005 | CVD defined as presence of recognized myocardial infarction, coronary insufficiency (unstable angina with demonstrated ischemic electrocardio-graphic changes), death due to coronary heart disease, or atherothrombotic stroke | - | Intake as g./ day | ADH1C | NR | ADH catalyses the reaction af ethanol to ethanal/ acetaldehyde, different polymorphsims in the ADH1 gene are linked to different rates of metabolising of alcohol. | No | NR |
| Hindy G et al, 2014 | MI cases were defined using codes 410 and I21 of the 9th and 10th revisions of the International Classification of Diseases (ICD9 and ICD10), and the stroke cases were defined using codes 430, 431, 434, and 436 of the ICD9, and codes I60, I61, I63, and I64 of the ICD10. | Registries | Low-medium-high g./ day | 9p21 locus | NR | This region is a significant genetic susceptibility locus for cardiovascular diseases. | No | Swedish Research Council, the Swedish, Heart and Lung Foundation, the Region Skåne, the Novo Nordic Foundation, the Albert Påhlsson Research Foundation, an equipment grant from the Knut |
| Sonestedt S et al, 2015 | Coronary heart disease was defined as ICD-9 codes 410– 414 (fatal or non-fatal myocardial infarction or death due to ischemic heart disease). Ischemic stroke was defined as ICD-9 code 434. | Registries, CT or autopsy. | Intake as g./ day. Usual dietary intakes were estimated using a modified diet history method specifically developed for MDCS | GRS-dyslipidaemia | NR | - | No | Swedish Medical Research Counci |
| Zhou A et al, 2019 | International Classification of Diseases (versions 9 and 10), as well as Classification of Interventions and Procedures | Medical records and registries | Coffee intake per day | CYP1A2 | CYP1A2 (rs762551) was genotyped with a call rate of 99.8% and a MAF of 0.27 | CYP1A2 encodes the CYP 450 1A2 enzyme which metabolises roughly 95% of caffeine in the liver, polymorphisms of this gene result in different activities in caffein metabolism. | No | Financially supported by the JJ Mason and HS WilliamsMemorial Foundation (CT23158) and the National Health and MedicalResearch Council, Australia |
| Articles evaluating at least two of the outcomes (CHD, MI, CVD or Stroke) | | | | | | | | |
| Livingstone M et al, 2021 | CVD mortality was identified using ICD-10 codes I05- I89. Incident MI (ST- Elevation MI and Non- ST- Elevation MI) and stroke (ischaemic, intracerebral haemorrhage and subarachnoid haemorrhage) | Health records and registries | RFS is a scoring system, to assess the amount of food consumed according to health guidelines. HDI is an index assessing intake of foods recommended by the WHO. MDS is a scoring system asseissing the adherence to a mediterranean diet. | GRS | NR | - | No | National Health and Medical Research Council Emerging Leadership Fellowship |
| Hellstrand S et al, 2016 | A coronary event was defined on the basis of codes 410–414 (fatal or non-fatal myocardial infarction or death due to ischemic heart disease) in the International Classification of Diseases, 9th Revision (ICD-9). Ischemic stroke was defined on the basis of code 434 (ICD-9) and diagnosed when computed tomography, magnetic resonance imaging, or autopsy could verify the infarction and/or exclude hemorrhage and non-vascular disease. | Register | The diet quality index includes six dietary components: the following cutoffs were used: saturated fat ≤14 E%, PUFA 5-10 E%, fish and shellfish ≥300 g/week, fiber ≥2.4 g/MJ, fruit and vegetables ≥400 g/day, and sucrose ≤10 E%. One point was given to the participants for each dietary component that reached the recommended intake level, and zero points were given if they were not within the recommended range. | GRS for LDL, HDL and Triglycerides | NR | - | Yes |  |
| Hellstrand S et al, 2014 | Coronary event was defined on the basis of codes 410–414 (fatal or nonfatal myocardial infarction or death due to ischemic heart disease) in the International Classification of Diseases, 9th Revision (ICD-9). Ischemic stroke was defined on the basis of ICD-9 code 434 and diagnosed when computed tomography, MRI, or autopsy could verify the infarction and/or exclude hemorrhage and nonvascular disease. If neither imaging nor autopsy was performed, the stroke was classified as unspecified. | Register | ALA (18:3n–3); long-chain n–3 PUFAs [EPA (20:5n–3), docosapentaenoic acid (DPA) (22:5n–3), and DHA (22:6n– 3)]; total n–3 PUFAs (ALA, EPA, DPA, and DHA); LA (C18:2n–6); total n–6 PUFAs [LA, g-LA (18:3n–6), and AA (20:4n–6)]; the ALA-to-LA intake ratio; and, the total n–3-to-total n–6 PUFA intake ratio. | FADS1 | NR | Desaturase enzymes of the Fatty acid desaturases (FADS) cluster regulate unsaturation of fatty acids through the introduction of double bonds between defined carbons of the fatty acyl chain. | Yes | Swedish Research Council (K2012-99X220018-01-3) |
| Zee R et al, 2007 | CVD was defined as nonfatal myocardial infarction, nonfatalischemic stroke, coronary revascularization, or death fromcardiovascular cause | Self-report, family report, registries and autopsy reports | Intake as mg./day Semiquantitative food-frequency questionnaire | MTHFR | Allele frequencies of 66.9% and 33.1% for the C and T alleles | Methylenetetrahydrofolate reductase plays an important role in the folate cycle and catalyses the conversion of 5,10-methylenetetrahydrofolate to 5-methyltetrahydrofolate, a co-substrate for homocysteine remethylation to methionine. | Yes | Donald W. Reynolds Founda-tion, the Leducq Foundation, and the Doris Duke CharitableFoundation |
| Heianza Y et al, 2020 | CVD was defined as fatal/ non-fatal stroke plus fatal/ non-fatal myocardial infarction, in accordance to ICD-10 classification of diseases. | Linkage to register data | 17 major food groups, within larger categories of healthy plant foods, less healthy plant foods, and animal foods. With positive scores, a score of 5 was given for the highest quintile category, following on through a score of 1 given for the lowest quintile category. With reverse scores, this pattern of scoring was inverted. Healthy plant foods received positive scores, whereas less healthy plant foods (such as refined grains, potatoes, and sweets) and animal foods received reverse scores. | GRS for stroke and myocardial infarction | NR | - | No | Supported by NIH grants from the National Heart, Lung, and |
| Do R et al, 2011 | Clinical characteristics of acute MI | Medical confirmation | Specific food pattern scores were derived from the food items using factor analysis. Three factors (dietary patterns) and were subjectively labeled as oriental (soy sauce, tofu, pickled foods, green leafy vegetables, eggs, and low sugar), western (eggs, meats, fried and salty foods, sugar, nuts, and desserts), and prudent (raw vegetables, fruits, green leafy vegetables, nuts, desserts, and dairy products) | 9p21 locus | Allele frequencies varied from 0.48 to 0.66 for the four genotyped SNPs | This region is a significant genetic susceptibility locus for cardiovascular diseases. | No | Heart and Stroke Foundation of Ontario |
| Miao L et al, 2017 | CHD can be defined as including typical ischemic symptoms, plus one or more electrocardiographic changes (ST-segment depression or elevation of ≥ 0.5 mm, T-wave inversion of ≥ 3 mm in ≥ 3 leads, or left bundle branch block), in addition to increases in cardiac markers, such as creatinine kinase-MB and troponin T. | Clinical examination - Medical confirmation | NR | MVK-MMAB | NR | Mevalonate kinase and methylmalonic aciduria both influence HDL levels. MVK plays a role in initial parts of cholesterol biosynthesis. For MMAB the exact metabolism by which it influences cholesterol is still unknown. | No | National Natural Science Foundation of China |
| Corella D et al, 2014 | CVD (Stroke, myocardial infarction or cardiovascular death) | Self-report. family and physicians reports, medical records, and National Death Index | 30 g./d Semiquantitative food-frequency questionnaire | LPL | LPL-rs13702 genotypes (minor allele frequency: 0.34 | LPL encodes lipoprotein lipase. LPL has the dual functions of triglyceride hydrolase and ligand/bridging factor for receptor-mediated lipoprotein uptake. Severe mutations that cause LPL deficiency result in type I hyperlipoproteinemia. | No | The Spanish Ministry of Economy and Innovation and the Fondo Europeo de Desarrollo Regional |
| Bergholdt H et al, 2015 | International Classification of Diseases, 8th and 10th IHD: 410-414, I20-I25; MI: 410, I21-I22 | Registries | Glasses/ week | LCT13910 | 76% and 24% for the T and C allele | LCT13910 is associated with lactase persistence/ non-persistence. Individuals with the genotypes TC/ TT are genetically predisposed to up-hold enzyme activity, whereas CC genotaype experience a natural down-regulation of the lactase enzyme during childhood, making it difficult to digest lactose in milk as adults. | No | Public and private |
| Zheng PF et al, 2021 | CAD was defined as significant coronary artery stenosis (≥ 50%) in at least one of the three major coronary arteries or their major branches. IS was defined according to International Classification of Diseases | Clinical examination and medical history collection | NR | SYTL3 and SLC22A3 | Genotypic frequencies of the SYTL3 rs2129209 and SLC22A3 rs539298 SNPs were different between controls and CAD/IS patients | SYTL3 encodes a protein of the family of peripheral membrane proteins that play a role in vesicular trafficking. SLC22A enables choline transmembrane transporter activity. Involved in acetylcholine transport; negative regulation of cholinergic synaptic transmission. | No | National Natural Science Foundation of China, Youthful Science Foundation of Guangxi Province, |
| Liu CX et al, 2022 | CHD was defined as typical clinical manifestations, myocardial enzymes, and electrocardiogram changes; coronary angiography revealing at least one major coronary artery, stenosis ≥50%. IS was defined as large atherosclerotic stroke and/or small-artery occlusive stroke and no CAD, hemorrhagic cerebral infarction, transient ischemic attack, and cerebral embolism. | Medical confirmation | Alcohol consumption was classified as 0 g/day, ≤25 g/day and >25 g/day. | EHBP1, TUBB and WWOX | The genotype and allele frequencies of the rs2278075, rs2710642, rs3130685, and rs2278075 were different between the CAD, IS and control groups. | TUBB gene encodes a beta tubulin protein. This protein forms a dimer with alpha tubulin and acts as a structural component of microtubules. WWOX encodes a member of the short-chain dehydrogenases/reductases (SDR) protein family. | No | National Natural Science Foundation of China, Youthful Science Foundation of Guangxi Province |
| Corella D et al, 2016 | CVD events and comprised a composite endpoint including myocardial infarction, stroke or cardiovascular death | Self-report. family and physicians reports, medical records, and National Death Index | NR | CLOCK | The minor allele frequency (MAF) for the G-variant allele was 0.38 | CLOCK is a gene involved in the endogenus circadian rhythm generation. In a complex interplay with other genes it works as a transcriptional factor generating the circadian rhythm. | No | NR |

NR no reported; CT: computarized tomography; MRI magnetic resonance imaging

GRS=Genetic Risk Score; Mediterranean diet score (MDS), plant-based diet score, recommended food score (RFS), healthy diet indicator (HDI), and prudent diet score

a The Nearest gene function was extracted from National Library of Medicine (US) Available from: https://www.ncbi.nlm.nih.gov/gene/

**Table S3.** Negative findings reported by the included studies.

| Dietary intake group | Author | Main findings regarding to the interaction evaluated |
| --- | --- | --- |
| Coronary heart disease | | |
| Macronutrients | Hartiala et al, 2012 (2) | No interaction was found with n-3 PUFA and PLA2G4C (rs12746200) on MI risk (p=0.30). Compared to AA homozygote subjects, carriers of AG/GG genotype who had high dietary n-3 PUFA intakes (≥1.02 g/day) have a lower MI odd with confidence intervals crossing the null value [OR 0.97 CI95% (0.77, 1.22)]. |
|  | Hellstrand et al, 2014 (3) | No interaction was observed between PUFA intake and FADS1 genotype on CVD risk. A borderline interaction was observed between the ALA/LA intake ratio and FADS1 genotype on CVD risk (p=0.06). In a stratified analysis, the ALA-to-LA intake ratio was inversely associated with CVD risk among the TT genotype carriers of rs174546 (HR for quintile 5 vs. quintile 1 = 0.72; 95% CI: 0.50, 1.04), none of the other genotypes showed association. |
|  | Gustavsson et al, 2016 (4) | No statistically significant interaction was found between FTO genotype and any macronutrients on CHD (fat, SFA, PUFA, Carbohydrate, sucrose, protein). However, in the sensitivity analyses, after the exclusion of subjects reporting diet change fat and SFA interacted with FTO genotype p 0.004 and 0.001, respectively. |
| Micronutrients | Zee et al, 2007 (5) | Folate or vitamins B intake did not significantly interact with MTHFR 677CT on the incidence of MI. Dietary exposures where evaluated as intake above or below median. |
| Food and food items categories | Bos M M et al, 2021(6) | Fish oil intake did not interacted with ApoE variants on CHD risk (p>0.10) |
|  | Bergholdt et al, 2015 (7) | Milk did not interact with the lactase LCT-13910 C/T genotype on the incidence of ischemic heart diseases and MI p=0.62 and p=0.30, respectively. |
|  | Huang et al, 2018 (8) | Marginal interaction between fried food intake and aldehyde dehydrogenase 2 (ALDH2) rs671 polymorphism, Relative Excess Risk due to Interaction (RERI) was 0.82(95% CI -0.01, 1.70) (p=0.052). Compared to carriers of GG genotype no consuming fried food, carriers of AA/AG genotypes with regular fried food intake had an increased odds of incident CHD [OR 1.86 (95%CI 1.07, 3.22); p=0.027]. In the stratified analysis an increased risk CHD was observed in the group of regular dessert intake for both genotypes (G/G and A/A A/G). |
|  | Virtanen et al, 2016 (9) | Neither cholesterol nor eggs intake significantly interacted with *APOE* variants on CHD risk p= 0.81 and p=0.34, respectively. In general, ApoE4 noncarriers had higher risk of CHD per additional egg consumption [HR 1.17 (CI 95% 0.85, 1.61)] and 100 mg/day of cholesterol intake [HR 1.04 (95% CI: 0.89, 1.22)]. |
| Other dietary components | Ebrahim et al, 2008 (10) | No evidence of interactions between moderate alcohol intake (≥3 units & < 21 units a week for women and <28 units for men) and ADH1C variants on CHD incidence (p>0.2) |
|  | Tolstrup et al, 2010 (11) | No evidence of interactions between alcohol consumption and ADH1B and ADH1C genotypes on CHD incidence (p=0.95) |
|  | Heidrich et al, 2007 (12) | No significant interactions were found among alcohol consumption, ADH1C genotype, and HDL cholesterol levels (p=0.07) |
|  | Younis et al, 2005 (13) | No interaction was reported between alcohol (drinkers vs abstainers) and ADH1C genotype on CHD (p=0.49). |
|  | Zheng PF et al, 2021(14) | The interaction of the SYTL3-SLC22A3 A-C-A-A-C-G drinking OR = 0.31, 95% CI = 0.14–0.67, (P < 0.01) decreased the risk of CHD. rs539298AG/GG genotypes and alcohol consumption interacted to reduced the risk of CAD ((14-17), P < 0.01) |
|  | Jensen et al, 2008 (18) | Interactions of alcohol consumption and CETP polymorphism were not significant neither in the Nurses' Health Study (NHS) (P=0.4) nor in the Health Professionals Follow-up Study (HPFS) (p=0.2). |
|  | Corella et al, 2010 (19) | No significant interaction was found between alcohol consumption (drinker vs non-drinker and non-drinker (0 g/day), moderate intake (<26.4 g alcohol/day for men and <13.2 g/day for women) and high intake (≥26.4 g alcohol/day for men and ≥13.2 g alcohol/day for women)) and CETP polymorphism. |
|  | Liu CX et al, 2022(17) | None of the evaluated haplotypes (rs2710642, rs10496099, rs3132584, rs3130685, rs2222896, and rs2278075) in EHBP1, TUBB, and WWOX) interacted with alcohol on CHD risk (p > 0.05). |
|  | Mukamal et al, 2009 (20) | Alcohol intake and paraoxonase 1(PON1) polymorphisms did not interacted significantly on MI risk in men Q192R (p=0.96) and L55M (p=0.83), neither in women Q192R (p= 0.06) and L55M genotype (p=0.11) |
|  | Chi et al, 2018 (21) | No significant interaction between alcohol and PLAG2G7variants (rs1805018 rs16874954 rs1805017, rs1051931) was found. |
|  | Zhou H et al, 2019 (22) | No significant interaction was found between alcohol intake and tissue factor pathway inhibitor-2 (TFPI-2) variants (rs34489123 rs59805398 rs4264 rs4271) on CHD. |
|  | Yiannakouris et al, 2014 (23) | No significant interaction was reported between GRS-CHD and MedDiet score on CHD. |
|  | Hellstrand et al, 2016 (24) | Neither GRS of HDL cholesterol, LDL cholesterol or triglycerides interacted with scores of diet quality indices (p>0.39). |
| Dietary scores/indices | Do et al, 2011 (25) | No significant interactions were found for the dietary risk score or for the oriental diet score and any of the 9p21 variants evaluated (rs10757274, rs2383206, rs10757278, rs1333049). However, it was reported an interaction between the rs2383206 variant and high consumption of foods classified as Western diet (eggs, meats, fried and salty foods, sugar, nuts and desserts) (p=0.028), which was not significant according to the corrected p-value threshold (p= 0.0024). |
|  | Trichopoulou et al, 2008 (26) | No significant interaction between adherence to the Mediterranean diet and GRS-MI was found (p=0.56). |
| Stroke | | |
| Food and food items categories | Zhao T et al 2019(27) | Consume of fruits and vegetables did not significantly interact with BCO2 or TRIB1 variants on stroke risk. P values between 0.49 and 0.95 |
| Other dietary components | Mukamal et al, 2005 (28) | The interaction between alcohol intake and ApoE genotype was not significant (p=0.08). However, authors highlighted that among ApoE4 negative carriers, alcohol drinkers had a decreased risk of stroke compared with abstainers. In addition, among ApoE4 positive carriers, alcohol drinkers had a higher risk than abstainers. |
|  | Luo et al, 2013 (29) | Alcohol intake did not interact significantly with IL-8 variant 781C/T (rs2227306) on stroke risk (p=0.93). |
|  | Zhang et al, 2019 (30) | Alcohol did not significantly interact with variants of PDE4D (rs966221, rs918592) or connexin 37 (CONNEXIN37) gene (rs1764391, rs1764390) p> 0.42. |
|  | Zheng X et al, 2020 (31) | Alcohol did not significantly interact with MTHFR variants rs1537514, rs3737967, rs484604 variatns on stroke risk, p values 0.172, 0.377, 0.532, respectively. |
|  | Zheng PF et al, 2021(14) | The interactions of the SYTL3-SLC22A3 A-CA-A-C-G-drinking OR = 0.37, 95% CI = 0.22–0.65, (p < 0.01) decreased the risk of IS. rs539298AG/GG genotypes and alcohol consumption interacted to reduced the risk of stroke (OR = 0.44, 95% CI = 0.30–0.65, P < 0.01) |
|  | Zhou YG et al, 2021(16) | Alcohol did not significantly interact with DGAT2 rs11236530 or DGAT2 rs3060 (PI > 0.05) on stroke risk. |
|  | Kamdee K et al, 2021(15) | Carriers of the IL‑6‑174 GG genotype who reported alcohol drinking had increased stroke risk compared with no drinkers OR=1.968, 95% CI=1.050‑3.688; p=0.035. Carriers of TNF‑α‑308 GG genotype who reported alcohol drinking had higher stroke risk compared with non drinkers OR=1.934, 95% CI=1.037‑3.608; (p=0.038). |
|  | Liu CX et al, 2022(17) | rs2710642A - rs10496099C haplotype interacted with alcohol to decreased the stroke risk, however rs2710642G-rs10496099C haplotype interacted with alcohol to increase stroke risk. (p<0.05) |
|  | Hellstrand et al, 2016(24) | In women, alcohol did not significantly interact with GRS-LDLc, nor with GRS-HDLc OR GRS-Tg on Stroke risk. p values between 0.12 and 0.55. |
| Dietary scores/indices | Livingstone et al, 2021 (32) | Moreover, in the UK Biobank, the RFS, HDI and MDS did not interact with GRS-CVD on stroke risk. |
|  | Heianza et al, 2021 (33) | PRS, RFS and PRS did not interacted with GRS-stroke on stroke risk (p >0.10) |
|  | Corella et al, 2016 (34) | Mediterranean diet did not interact with Circadian locomotor output cycles kaput (CLOCK) variant rs4580704 on the risk of stroke (p=0.439). |
| CVD | | |
| Food and food items | Zhou H et al, 2019(22) and Zhou A et al, 2019 (35) | Coffee intake did not significantly interact with CYP1A2 or a GRS-Caffeine on CVD risk, p>0.53 |
| Other dietary components | Djoussé et al, 2005(36) | No statistically significant interactions between drinking status and the rs693482 and rs698 SNPs of the ADH1C gene on CVD odds. |
| Dietary scores/indices | Hellstrand et al, 2016 (24) | Diet quality did not interact significantly with none of the GRS evaluated GRS-LDL cholesterol p=0.39, GRS-HDL cholesterol p= 0.85, and GRS-triglycerides p= 0.86 (61). |
| CHD, CVD, OR stroke | | |
| Other dietary components | Miao et al, 2017 (37) | No interaction between alcohol and MVK and MMAB variants (rs7134594, rs877710 and rs9593) on CHD and stroke was found. |
| Dietary scores/indices | Heianza et al, 2020 (33) | No significant interaction between Healthful plant-based diet index and GRS of MI or Stroke risk was found, p=0.66 and p=0.63. |

**Table S4:** Interaction gene environmental methodological quality assessments of included studies.

| First author/Year | Interaction as primary study goal | Formal test for interaction | Correction for multiple testing | Correction for ethnicity | Hardy-Weinberg equilibrium | Group similarity at baseline tested | Sample size | Sufficient details of study procedure stated | Score | Quality classification |
| --- | --- | --- | --- | --- | --- | --- | --- | --- | --- | --- |
| Allayee H et al, 2008 | 1 | 1 | -1 | 1 | -1 | 1 | 0 | 1 | 3 | Intermediate |
| Bergholdt H et al, 2015 | -1 | 1 | 0 | 1 | 1 | 1 | 1 | 1 | 5 | Intermediate |
| Bos M M et al, 2021 | 1 | 1 | 0 | -1 | 1 | 1 | 1 | 1 | 5 | Intermediate |
| Chen H et al, 2018 | 1 | 1 | -1 | 1 | 1 | 1 | -1 | -1 | 2 | Intermediate |
| Chen Q F et al, 2017 | 1 | 1 | 0 | 1 | 1 | 1 | 0 | 1 | 6 | High |
| Chen Z et al, 2015 | 1 | 1 | -1 | 1 | 1 | 1 | -1 | -1 | 2 | Intermediate |
| Chi Y et al, 2018 | 1 | 1 | 0 | -1 | 1 | 1 | 1 | 1 | 5 | Intermediate |
| Corella D et al, 2010 | 0 | 1 | -1 | 1 | 1 | 1 | 0 | 1 | 4 | Intermediate |
| Corella D et al, 2014 | 1 | 1 | -1 | 1 | 1 | 1 | 1 | 1 | 6 | High |
| Corella D et al, 2016 | 1 | 1 | -1 | 1 | 1 | 1 | 1 | 1 | 6 | High |
| Cornelis C et al, 2007 | 1 | 1 | -1 | 1 | 1 | 1 | 0 | 1 | 5 | Intermediate |
| Cornelis M et al, 2006 | 1 | 1 | 1 | -1 | 1 | 1 | 0 | 1 | 5 | Intermediate |
| Ding Y et al, 2016 | 1 | 1 | -1 | 1 | 1 | 1 | 0 | -1 | 3 | Intermediate |
| Djousse L et al, 2005 | 1 | 1 | -1 | 1 | 1 | 1 | 0 | -1 | 3 | Intermediate |
| Do R et al, 2011 | 1 | 1 | 1 | 1 | 1 | 1 | 1 | 1 | 8 | High |
| Ebrahim S et al, 2008 | 1 | 1 | -1 | 1 | 1 | 1 | 0 | 1 | 5 | Intermediate |
| Fumeron F et al, 1995 | 1 | 1 | -1 | 1 | 1 | 1 | -1 | 1 | 4 | Intermediate |
| Gao X et al, 2006 | 1 | 1 | -1 | 1 | -1 | 1 | -1 | 1 | 2 | Intermediate |
| Gustavsson J et al, 2016 | 1 | 1 | -1 | 1 | 1 | 1 | 1 | -1 | 4 | Intermediate |
| Hartiala J et al, 2012 | 1 | 1 | -1 | 1 | 1 | 1 | 0 | 1 | 5 | Intermediate |
| Heianza Y et al, 2020 | 0 | 1 | 0 | 1 | -1 | 1 | 1 | 1 | 4 | Intermediate |
| Heidrich J et al, 2007 | 1 | 1 | -1 | 1 | 1 | -1 | 0 | -1 | 1 | Poor |
| Hellstrand S et al, 2014 | 1 | 1 | -1 | 1 | 1 | 1 | 1 | 1 | 6 | High |
| Hellstrand S et al, 2016 | 0 | 1 | -1 | 1 | 1 | 1 | 1 | 1 | 5 | Intermediate |
| Hindy G et al, 2014 | 1 | 1 | -1 | 1 | 1 | 1 | 1 | 1 | 6 | High |
| Hines L et al, 2001 | 1 | 1 | -1 | -1 | 1 | -1 | 0 | 1 | 1 | Poor |
| Huang L et al, 2018 | 1 | 1 | 1 | 1 | 1 | 1 | -1 | 1 | 6 | High |
| Jensen M et al, 2008 | 1 | 1 | -1 | 1 | 1 | 1 | 1 | 1 | 6 | High |
| Juan J. et al, 2017 | 1 | 1 | -1 | 1 | 1 | 1 | 0 | 1 | 5 | Intermediate |
| Kamdee K et al, 2021 | 1 | 1 | -1 | -1 | 1 | 1 | 0 | 1 | 3 | Intermediate |
| Li J et al, 2020 | 1 | 1 | 1 | 0 | 1 | 1 | 1 | 1 | 7 | High |
| Liu CX et al, 2022 | 1 | 1 | 1 | -1 | 1 | 1 | 0 | 1 | 5 | Intermediate |
| Liu F et al, 2015 | 1 | 1 | -1 | 1 | 1 | 1 | 0 | -1 | 3 | Intermediate |
| Liu TY et al, 2020 | 1 | 1 | 0 | 1 | 1 | 1 | 1 | 0 | 6 | High |
| Livingstone M. et al, .2021 | 0 | 1 | 0 | 0 | -1 | 1 | 1 | 1 | 3 | Intermediate |
| Luo S et al, 2013 | 1 | 1 | 0 | 0 | 1 | 1 | 0 | 1 | 5 | Intermediate |
| Mehlig K et al, 2014 | 1 | 1 | -1 | 1 | 1 | 1 | 0 | 1 | 5 | Intermediate |
| Miao L et al, 2017 | 1 | 1 | 1 | 1 | 1 | 1 | 0 | 1 | 7 | High |
| Mukamal K et al, 2005 | 1 | 1 | 0 | -1 | -1 | 1 | 0 | 1 | 2 | Intermediate |
| Mukamal K et al, 2009 | 1 | 1 | -1 | -1 | 1 | 1 | 0 | 1 | 3 | Intermediate |
| Sonestedt E et al, 2015 | 1 | 1 | -1 | 1 | 1 | 1 | 1 | 1 | 6 | High |
| Song J et al, 2019 | 1 | 1 | -1 | 1 | 1 | 1 | 1 | 1 | 6 | High |
| Tolstrup J et al, 2009 | 1 | 1 | -1 | 1 | 1 | 1 | 1 | 1 | 6 | High |
| Tolstrup J et al, 2010 | 1 | 1 | -1 | 1 | 1 | 1 | 0 | 1 | 5 | Intermediate |
| Trichopoulou A et al, 2008 | 1 | 1 | -1 | 1 | -1 | 1 | -1 | 1 | 2 | Intermediate |
| Virtanen J et al, 2016 | 1 | 1 | -1 | 1 | -1 | 1 | 0 | 1 | 3 | Intermediate |
| Wang F et al, 2019 | 1 | 1 | 1 | 0 | 1 | 1 | 0 | 1 | 6 | High |
| Yang S et al, 2020 | 1 | 1 | 1 | 1 | 1 | 1 | 0 | 1 | 7 | High |
| Yiannakouris N et al, 2014 | 1 | 1 | 0 | 1 | -1 | 1 | 0 | 1 | 4 | Intermediate |
| Younis J et al, 2005 | 1 | 1 | -1 | 1 | 1 | 1 | 0 | -1 | 3 | Intermediate |
| Zee R et al, 2007 | 1 | 1 | -1 | 1 | 1 | 1 | 1 | 1 | 6 | High |
| Zhang L et al, 2019 | 0 | 1 | 1 | 1 | 1 | 1 | 0 | 1 | 6 | High |
| Zhao T et al, 2019 | 0 | 1 | 0 | 0 | 1 | 1 | 0 | 0 | 3 | Intermediate |
| Zheng PF et al, 2021 | 1 | 1 | 1 | 0 | 1 | 1 | 0 | 1 | 6 | High |
| Zheng XZ et al, 2020 | 0 | 1 | 0 | 1 | 1 | 1 | 0 | 1 | 5 | Intermediate |
| Zheng Y et al, 2017 | 1 | 1 | -1 | 1 | 1 | 1 | 0 | -1 | 3 | Intermediate |
| Zhou A et al, 2019 | 1 | 1 | -1 | 1 | 1 | 1 | 1 | 1 | 6 | High |
| Zhou YG et al, 2021 | 1 | 1 | 1 | 0 | 1 | 1 | 1 | 1 | 7 | High |
| Zhou H et al, 2019 | 1 | 1 | 0 | 0 | 1 | 1 | 0 | 1 | 5 | Intermediate |

Methodological quality of GXE interaction research. Dietrich, Stefan, et al. "Gene‐lifestyle interaction on risk of type 2 diabetes: a systematic review." *Obesity Reviews* 20.11 (2019): 1557-1571.

**Table S5** Checklist of the Synthesis without meta-analysis (SWiM) in systematic reviews: reporting guideline

| SWIM Reporting Item | Item description | Page in the manuscript |
| --- | --- | --- |
| 1 Grouping studies for synthesis | 1a) Provide a description of, and rationale for, the groups used in the synthesis (eg, groupings of populations, interventions, outcomes, study design) | Page 10 |
|  | 1b) Detail and provide rationale for any changes made subsequent to the protocol in the groups used in the synthesis |  |
| 2 Describe the standardised metric and Describe the standardised metric for each outcome. Explain why the metric(s) was chosen and transformation methods used | describe any methods used to transform the intervention effects, as reported in the study, to the standardised metric, citing any methodological guidance consulted | No transformation was performed |
| 3 Describe the synthesis methods | Describe and justify the methods used to synthesise the effects for each outcome when it was not possible to undertake a meta-analysis of effect estimates | Page 11 |
| 4 Criteria used to prioritise results for summary and synthesis | Where applicable, provide the criteria used, with supporting justification, to select the particular studies, or a particular study, for the main synthesis or to draw conclusions from the synthesis (eg, based on study design, risk of bias assessments, directness in relation to the review question) | Page 10 |
| 5 Investigation of heterogeneity in reported effects | State the method(s) used to examine heterogeneity in reported effects when it was not possible to undertake a meta-analysis of effect estimates and its extensions to investigate heterogeneity | Page 11 |
| 6 Certainty of evidence | Describe the methods used to assess the certainty of the synthesis findings | Page 10 |
| 7 Data presentation methods | Describe the graphical and tabular methods used to present the effects (eg, tables, forest plots, harvest plots) | Page 11 |
|  | Specify key study characteristics (eg, study design, risk of bias) used to order the studies, in the text and any tables or graphs, clearly referencing the studies included |  |
| Results |  |  |
| 8 Reporting results | For each comparison and outcome, provide a description of the synthesised findings and the certainty of the findings. Describe the result in language that is consistent with the question the synthesis addresses, and indicate which studies contribute to the synthesis | Pages 11-23 |
| Discussion |  |  |
| 9 Limitations of the synthesis | Report the limitations of the synthesis methods used and/or the groupings used in the synthesis and how these affect the conclusions that can be drawn in relation to the original review question | Page 28 |

References

1. Wardlaw GM, Byrd-Bredbenner C. Wardlaw's perspectives in nutrition: McGraw-Hill; 2013.

2. Hartiala J, Gilliam E, Vikman S, Campos H, Allayee H. Association of PLA2G4A with myocardial infarction is modulated by dietary PUFAs. American Journal of Clinical Nutrition. 2012;95(4):959-65.

3. Hellstrand S, Ericson U, Gullberg B, Hedblad B, Orho-Melander M, Sonestedt E. Genetic variation in FADS1 has little effect on the association between dietary PUFA intake and cardiovascular disease. Journal of Nutrition. 2014;144(9):1356-63.

4. Gustavsson J, Mehlig K, Leander K, Berg C, Tognon G, Strandhagen E, et al. FTO gene variation, macronutrient intake and coronary heart disease risk: a gene-diet interaction analysis. European Journal of Nutrition. 2016;55(1):247-55.

5. Zee RY, Mora S, Cheng S, Erlich HA, Lindpaintner K, Rifai N, et al. Homocysteine, 5,10-methylenetetrahydrofolate reductase 677C>T polymorphism, nutrient intake, and incident cardiovascular disease in 24,968 initially healthy women. Clinical Chemistry. 2007;53(5):845-51.

6. Bos MM, de Vries L, Rensen PC, Willems van Dijk K, Blauw GJ, van Heemst D, et al. Apolipoprotein E genotype, lifestyle and coronary artery disease: Gene-environment interaction analyses in the UK Biobank population. Atherosclerosis. 2021;328:33-7.

7. Bergholdt HK, Nordestgaard BG, Varbo A, Ellervik C. Milk intake is not associated with ischaemic heart disease in observational or Mendelian randomization analyses in 98,529 Danish adults. International Journal of Epidemiology. 2015;44(2):587-603.

8. Huang L, Cai X, Lian F, Zhang L, Kong Y, Cao C, et al. Interactions between ALDH2 rs671 polymorphism and lifestyle behaviors on coronary artery disease risk in a Chinese Han population with dyslipidemia: A guide to targeted heart health management. Environ Health Prev Med. 2018;23(1):29.

9. Virtanen JK, Mursu J, Virtanen HEK, Fogelholm M, Salonen JT, Koskinen TT, et al. Associations of egg and cholesterol intakes with carotid intima-media thickness and risk of incident coronary artery disease according to apolipoprotein e phenotype in men: the Kuopio Ischaemic Heart Disease Risk Factor Study. American journal of clinical nutrition. 2016;103(3):895‐901.

10. Ebrahim S, Lawlor DA, Shlomo YB, Timpson N, Harbord R, Christensen M, et al. Alcohol dehydrogenase type 1C (ADH1C) variants, alcohol consumption traits, HDL-cholesterol and risk of coronary heart disease in women and men: British Women's Heart and Health Study and Caerphilly cohorts. Atherosclerosis. 2008;196(2):871-8.

11. Tolstrup JS, Hansen JL, Gronbaek M, Vogel U, Tjonneland A, Joensen AM, et al. Alcohol drinking habits, alcohol dehydrogenase genotypes and risk of acute coronary syndrome. Scandinavian journal of public health. 2010;38(5):489-94.

12. Heidrich J, Wellmann J, Doring A, Illig T, Keil U. Alcohol consumption, alcohol dehydrogenase and risk of coronary heart disease in the MONICA/KORA-Augsburg cohort 1994/1995-2002. European Journal of Cardiovascular Prevention & Rehabilitation. 2007;14(6):769-74.

13. Younis J, Cooper JA, Miller GJ, Humphries SE, Talmud PJ. Genetic variation in alcohol dehydrogenase 1C and the beneficial effect of alcohol intake on coronary heart disease risk in the Second Northwick Park Heart Study. Atherosclerosis. 2005;180(2):225-32.

14. Zheng PF, Yin RX, Cao XL, Chen WX, Wu JZ, Huang F. Effect of SYTL3-SLC22A3 Variants, Their Haplotypes, and G × E Interactions on Serum Lipid Levels and the Risk of Coronary Artery Disease and Ischaemic Stroke. Front Cardiovasc Med. 2021;8:713068.

15. Kamdee K, Panadsako N, Mueangson O, Nuinoon M, Janwan P, Poonsawat W, et al. Promoter polymorphism of TNF-alpha (rs1800629) is associated with ischemic stroke susceptibility in a southern Thai population. Biomed. 2021;15(3):78.

16. Zhou YG, Yin RX, Huang F, Wu JZ, Chen WX, Cao XL. DGAT2-MOGAT2 SNPs and Gene-Environment Interactions on Serum Lipid Profiles and the Risk of Ischemic Stroke. Front. 2021;8:685970.

17. Liu CX, Yin RX, Cao XL, Shi ZH, Huang F, Wei BL, et al. EHBP1, TUBB, and WWOX SNPs, Gene-Gene and Gene-Environment Interactions on Coronary Artery Disease and Ischemic Stroke. Frontiers in Genetics. 2022;13.

18. Jensen MK, Mukamal KJ, Overvad K, Rimm EB. Alcohol consumption, TaqIB polymorphism of cholesteryl ester transfer protein, high-density lipoprotein cholesterol, and risk of coronary heart disease in men and women. European Heart Journal. 2008;29(1):104-12.

19. Corella D, Carrasco P, Amiano P, Arriola L, Chirlaque MD, Huerta JM, et al. Common cholesteryl ester transfer protein gene variation related to high-density lipoprotein cholesterol is not associated with decreased coronary heart disease risk after a 10-year follow-up in a Mediterranean cohort: Modulation by alcohol consumption. Atherosclerosis. 2010;211(2):531-8.

20. Mukamal KJ, Pai JK, Jensen MK, Rimm EB. Paraoxonase 1 polymorphisms and risk of myocardial infarction in women and men. Circulation Journal. 2009;73(7):1302-7.

21. Chi Y, Shi C, Zhang X, Xi Y. Interaction between nonsynonymous polymorphisms in PLA2G7 gene and smoking on the risk of coronary heart disease in a Chinese population. Journal of Thrombosis and Thrombolysis. 2018;46(1):125-30.

22. Zhou H, Che Y, Fu X, Wei H, Gao X, Chen Y, et al. Interaction between tissue factor pathway inhibitor-2 gene polymorphisms and environmental factors associated with coronary atherosclerosis in a Chinese Han. Journal of Thrombosis and Thrombolysis. 2019;47(1):67-72.

23. Yiannakouris N, Katsoulis M, Trichopoulou A, Ordovas JM, Trichopoulos D. Additive influence of genetic predisposition and conventional risk factors in the incidence of coronary heart disease: a population-based study in Greece. BMJ Open. 2014;4(2):e004387.

24. Hellstrand S, Ericson U, Schulz CA, Drake I, Gullberg B, Hedblad B, et al. Genetic susceptibility to dyslipidemia and incidence of cardiovascular disease depending on a diet quality index in the Malmo Diet and Cancer cohort. Genes & Nutrition. 2016;11:20.

25. Do R, Xie C, Zhang X, Mannisto S, Harald K, Islam S, et al. The effect of chromosome 9p21 variants on cardiovascular disease may be modified by dietary intake: evidence from a case/control and a prospective study. PLoS Medicine / Public Library of Science. 2011;8(10):e1001106.

26. Trichopoulou A, Yiannakouris N, Bamia C, Benetou V, Trichopoulos D, Ordovas JM. Genetic predisposition, nongenetic risk factors, and coronary infarct. Archives of Internal Medicine. 2008;168(8):891-6.

27. Zhao TY, Li Z, Lei S, Huang L, Yang L. Associations for BCO2, PCSK9, and TR1B1 Polymorphism and Lifestyle Factors with Ischemic Stroke: A Nested Case-Control Study. Yonsei Med J. 2019;60(7):659-66.

28. Mukamal KJ, Chung H, Jenny NS, Kuller LH, Longstreth WT, Jr., Mittleman MA, et al. Alcohol use and risk of ischemic stroke among older adults: the cardiovascular health study. Stroke. 2005;36(9):1830-4.

29. Luo S, Wang F, Li Z, Deng J. Effect of the +781C/T polymorphism in the interleukin-8 gene on atherosclerotic cerebral infarction, and its interaction with smoking and drinking. PLoS ONE. 2013;8(11).

30. Zhang L, Ding R, Kuang P, Wang L, Deng H, Xiong Q, et al. Interaction between CONNEXIN37 and PDE4D gene polymorphisms with susceptibility to ischemic stroke in Chinese population. Experimental Biology and Medicine. 2019;244(18):1642-7.

31. Zheng XZ, Bian XL, Sun ZH, Wang HD. Interaction Between Methylenetetrahydrofolate Reductase (MTHFR) Gene Polymorphisms and Environment with Susceptibility to Ischemic Stroke in Chinese Population. Ann. 2020;23(4):491-5.

32. Livingstone KM, Abbott G, Bowe SJ, Ward J, Milte C, McNaughton SA. Diet quality indices, genetic risk and risk of cardiovascular disease and mortality: a longitudinal analysis of 77 004 UK Biobank participants. BMJ Open. 2021;11(4):e045362.

33. Heianza Y, Zhou T, Sun D, Hu FB, Manson JE, Qi L. Genetic susceptibility, plant-based dietary patterns, and risk of cardiovascular disease. American Journal of Clinical Nutrition. 2020;112(1):220-8.

34. Corella D, Asensio EM, Coltell O, Sorli JV, Estruch R, Martinez-Gonzalez MA, et al. CLOCK gene variation is associated with incidence of type-2 diabetes and cardiovascular diseases in type-2 diabetic subjects: dietary modulation in the PREDIMED randomized trial. Cardiovascular Diabetology. 2016;15:4.

35. Zhou A, Hypponen E. Long-term coffee consumption, caffeine metabolism genetics, and risk of cardiovascular disease: A prospective analysis of up to 347,077 individuals and 8368 cases. American Journal of Clinical Nutrition. 2019;109(3):509-16.

36. Djousse L, Levy D, Herbert AG, Wilson PW, D'Agostino RB, Cupples LA, et al. Influence of alcohol dehydrogenase 1C polymorphism on the alcohol-cardiovascular disease association (from the Framingham Offspring Study). American Journal of Cardiology. 2005;96(2):227-32.

37. Miao L, Yin RX, Huang F, Chen WX, Cao XL, Wu JZ. The effect of MVK-MMAB variants, their haplotypes and GxE interactions on serum lipid levels and the risk of coronary heart disease and ischemic stroke. Oncotarget. 2017;8(42):72801-17.
